# Transient expression of ZBTB32 in anti-viral CD8^+^ T cells limits the magnitude of the effector response and the generation of memory

**DOI:** 10.1371/journal.ppat.1006544

**Published:** 2017-08-21

**Authors:** Hyun Mu Shin, Varun N. Kapoor, Gwanghun Kim, Peng Li, Hang-Rae Kim, M. Suresh, Susan M. Kaech, E. John Wherry, Liisa K. Selin, Warren J. Leonard, Raymond M. Welsh, Leslie J. Berg

**Affiliations:** 1 Dept of Pathology, University of Massachusetts Medical School, Worcester, Massachusetts, United States of America; 2 Department of Anatomy and Cell Biology, Department of Biomedical Sciences, and BK21Plus Biomedical Science Project, Seoul National University College of Medicine, Seoul, Republic of Korea; 3 Laboratory of Molecular Immunology and the Immunology Center, National Heart, Lung, and Blood Institute (NHLBI), National Institutes of Health, Bethesda, Maryland, United States of America; 4 Department of Pathobiological Sciences, School of Veterinary Medicine, University of Wisconsin-Madison, Madison, Wisconsin, United States of America; 5 Department of Immunobiology, Yale University School of Medicine, New Haven, Connecticut, United States of America; 6 Department of Microbiology and Institute for Immunology, University of Pennsylvania Perelman School Medicine, Philadelphia, Pennsylvania, United States of America; Nationwide Children's Hospital, UNITED STATES

## Abstract

Virus infections induce CD8^+^ T cell responses comprised of a large population of terminal effector cells and a smaller subset of long-lived memory cells. The transcription factors regulating the relative expansion versus the long-term survival potential of anti-viral CD8^+^ T cells are not completely understood. We identified ZBTB32 as a transcription factor that is transiently expressed in effector CD8^+^ T cells. After acute virus infection, CD8^+^ T cells deficient in ZBTB32 showed enhanced virus-specific CD8^+^ T cell responses, and generated increased numbers of virus-specific memory cells; in contrast, persistent expression of ZBTB32 suppressed memory cell formation. The dysregulation of CD8^+^ T cell responses in the absence of ZBTB32 was catastrophic, as *Zbtb32*^*-/-*^ mice succumbed to a systemic viral infection and showed evidence of severe lung pathology. We found that ZBTB32 and Blimp-1 were co-expressed following CD8^+^ T cell activation, bound to each other, and cooperatively regulated Blimp-1 target genes *Eomes* and *Cd27*. These findings demonstrate that ZBTB32 is a key transcription factor in CD8^+^ effector T cells that is required for the balanced regulation of effector versus memory responses to infection.

## Author summary

CD8^+^ T lymphocytes are essential for immune protection against viruses. In response to an infection, these cells are activated, proliferate, and generate antiviral effector cells that eradicate the infection. Following this, the majority of these effector cells die, leaving a small subset of long-lived virus-specific memory T cells. Our study identifies a transcription factor, ZBTB32, that is required for the regulation of CD8^+^ T cell responses. In its absence, antiviral CD8^+^ T cell numbers increase to abnormally high levels, and generate an overabundance of memory T cells. When this dysregulated response occurs following infection with a virus that cannot be rapidly eliminated by the immune system, the infected animals die from immune-mediated tissue damage, indicating the importance of this pathway.

## Introduction

The anti-viral CD8^+^ T cell response has been the topic of intense investigation over recent years, beginning with early ground-breaking studies demonstrating that, at early times post-infection, effector cells destined to die could be distinguished from those destined to populate the long-lived memory pool [1]. Molecular analysis of these subsets has revealed complex networks of transcription factors regulating the numbers, the phenotypes, and the survival potential of antiviral CD8^+^ T cells in models of both acute and chronic infections [2–4]. Contributing to this complexity, lineage-tracing experiments showed that the clonal responses of individual CD8^+^ T cells activated *in vivo* exhibited dramatic heterogeneity, and further, that this heterogeneity was already apparent at early times post-infection [5,6]. These studies also showed an inverse correlation between T cell family size at the peak of the response and the expression of memory T cell markers. Furthermore, mathematical modeling of these data indicated a linear pattern of differentiation with memory precursor cells arising first, undergoing limited proliferation, followed by a small number of these cells undergoing massive expansion to comprise the majority of the terminal effector population. Single-cell RNA-seq data have elaborated on these findings, identifying subpopulations of activated CD8^+^ T cells that show effector-like and memory-like gene expression profiles that can be seen as early as the first cell division [7].

While the source of the variability in clonal T cell responses is not currently known, one likely possibility is a variation in local concentrations of antigen and inflammatory cytokines, as these signals have been shown to regulate the magnitude of antiviral CD8^+^ T cell responses and the generation of memory cells [8–12]. Thus, transcription factors that are upregulated by a combination of TCR and inflammatory cytokine signals would be likely candidates to contribute to the regulation of clonal T cell responses. One such transcription factor is Blimp-1 (encoded by *Prdm1*), which has a critical role in promoting the terminal differentiation of CD8^+^ effector T cells [13,14]. We have recently shown that Blimp-1 acts as an epigenetic regulator and enhances the numbers of short-lived effector cells, while suppressing the development of memory-precursor CD8^+^ T cells [15]. Here we identify a second transcription factor, ZBTB32/ROG, as being rapidly upregulated in anti-viral CD8^+^ T cells in a TCR- and inflammatory cytokine-dependent manner. ZBTB32 belongs to the POK (Poxviruses and Zinc-finger (POZ) and Krüppel) family of proteins, most of which are transcriptional repressors, such as PLZF (Promyelocytic leukemia Zn finger protein) and BCL6 (B cell lymphoma-6) [16]. ZBTB32 is expressed in T and B cells upon activation [17–22], and has been shown to inhibit IL-4 gene activation by recruiting histone deacetylase (HDAC) 1 and 2 [19]. *Zbtb32*-deficient CD4^+^ T cells showed enhanced proliferation and cytokine production following *in vitro* stimulation [18,20,21]. Consistent with this, overexpression of ZBTB32 in BDC2.5 CD4^+^ T cells suppressed T cell proliferation and cytokine production [23]. *Zbtb32*-deficient CD8^+^ T cells were found to have enhanced responses to MCMV infection, whereas the opposite effect was observed in NK cells responding to the infection [24]. In addition, studies of plasma cell differentiation suggested that ZBTB32 and Blimp-1 form a complex to regulate the *Ciita* and *H2* genes during this process [22]. Recently, ZBTB32 was shown to be a negative regulator of memory B cell recall responses [25]. Nonetheless, the function of ZBTB32 in regulating anti-viral CD8^+^ T cell responses *in vivo* is currently not known.

Here we addressed the function of ZBTB32 in CD8^+^ T cell responses to both acute and chronic virus infections. We found that mice deficient in *Zbtb32* generated an enhanced anti-viral CD8^+^ T cell response during acute virus infection and had increased memory CD8^+^ T cell populations; conversely the sustained expression of *Zbtb32* in virus-specific CD8^+^ T cells dampened the anti-viral T cell response. Molecular analysis demonstrated that *Zbtb32* induction following TCR plus cytokine stimulation resulted from STAT1, STAT4 or STAT5 binding to the regulatory region of the *Zbtb32* locus, and that later in the response, *Zbtb32* was repressed by Blimp-1. Finally, we showed that ZBTB32 and Blimp-1 acted cooperatively to mediate repressive chromatin modifications at key target genes during the peak of the anti-viral CD8^+^ T cell response, thereby dictating the magnitude of the response and the numbers of memory T cells generated.

## Results

### *Zbtb32* is a direct target of STAT1, 4 or 5 in CD8^+^ T cells

**Fig 1:**
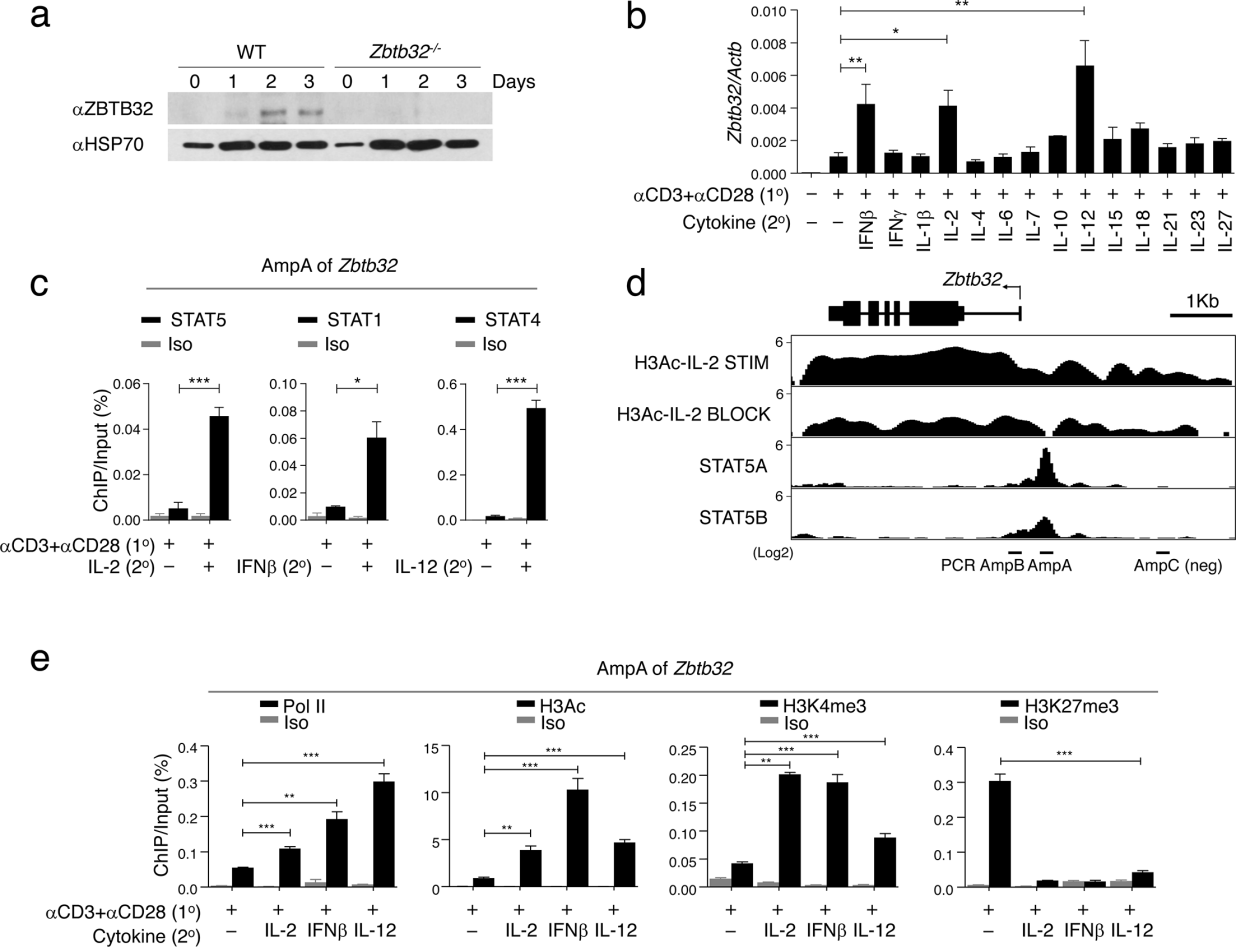
Inflammatory cytokines induce maximal transcription of *Zbtb32*. (a) Purified CD8^+^ T cells from WT or *Zbtb32*^*-/-*^ mice were activated with αCD3/CD28 *in vitro* as indicated, then harvested for immunoblotting with rabbit αZBTB32 antibody. Data are representative of two independent experiments.(b) CD8^+^ T cells were pre-activated with or without αCD3/CD28 for 24h (1^o^) and then stimulated with indicated cytokines for 1 day (2^o^) and *Zbtb32* mRNA was analyzed by Q-PCR relative to *Actb* mRNA. (c) Pre-activated (1^o^) WT CD8^+^ T cells were stimulated with or without (2^o^) IL-2 (left), IFN-β (middle) or IL-12 (right) for 30 min for ChIP assay. Binding of STAT-5 (left), STAT-1 (middle) or STAT-4 (right) at AmpA (target regions) of *Zbtb32* is depicted. (d) ChIP-seq analysis of STAT5A and STAT5B binding [26] and histone H3-acetylation (H3Ac) at *Zbtb32*. H3Ac ChIP-seq was performed on naïve OT-I CD8^+^ T cells stimulated with αCD3/CD28 *in vitro* for three days with IL-2 (IL-2 STIM) or αCD25/αCD122/αIL-2 (IL-2 BLOCK). Binding enrichment for STAT5A/B correlates with significant changes in H3Ac in IL-2 STIM versus IL-2 BLOCK. The positions of AmpA and B (target regions) and AmpC (non-specific control) used in panels B and D are shown on the *Zbtb32* locus. (e) Pre-activated (1^o^) WT CD8^+^ T cells were stimulated *in vitro* with or without IL-2, IFNβ and IL-12 (2^o^) for 3 hours. Chromatin was prepared for Pol II, H3Ac, H3K3me3 and H3K27me3 ChIP analysis compared to isotype control (Iso). ChIP eluates were amplified by Q-PCR for the indicated regions of the *Zbtb32* gene locus (AmpA).Data (b,c and e) are compilations from three independent experiments; error bars represent SEM.

In CD8^+^ T cells, ZBTB32 was up-regulated upon stimulation with α-CD3/CD28 (Fig 1A). We then examined the cytokines involved in the induction of *Zbtb32* mRNA. Primary CD8^+^ T cells were pre-activated with α-CD3/CD28, and then cultured in a panel of cytokines (Fig 1B). *Zbtb32* mRNA was up-regulated in response to IL-2, IFNβ and IL-12 (Fig 1B). Moreover, Chromatin immunoprecipitation (ChIP) assays at the *Zbtb32* locus revealed that IL-2, IFNβ and IL-12 could induce STAT5, STAT1 and STAT4 binding, respectively, to the *Zbtb32* proximal promoter (AmpA) and the 5’ UTR region (AmpB), but not to a non-specific region of the locus (AmpC) (Fig 1C and 1D and S1A Fig). Genome-wide STAT5 ChIP-seq analysis [26] showed that both dimeric and tetrameric forms of STAT5A and STAT5B bound to the regulatory region of *Zbtb32* upon IL-2 stimulation and was associated with increased H3-Ac modification (Fig 1D). Moreover, STAT5 binding was associated with active gene transcription and histone modifications, based on increased RNA polymerase II (Pol II) binding and high amounts of permissive H3-Ac, H3K4me3, but low amounts of repressive H3K27me3 modifications, compared to a non-specific region of the locus (AmpC) (Fig 1E and S1B Fig). STAT1 and 4 binding were also associated with active gene transcription and histone modifications at STAT binding regions (Fig 1E and S1B Fig). These findings demonstrated that IL-2, IFNβ and IL-12 signaling could each initiate STAT binding, leading to the establishment of an active chromatin state at the *Zbtb32* locus in CD8^+^ T cells.

### Increased CD8^+^ effector T cell responses in *Zbtb32*^*-/-*^ mice

**Fig 2:**
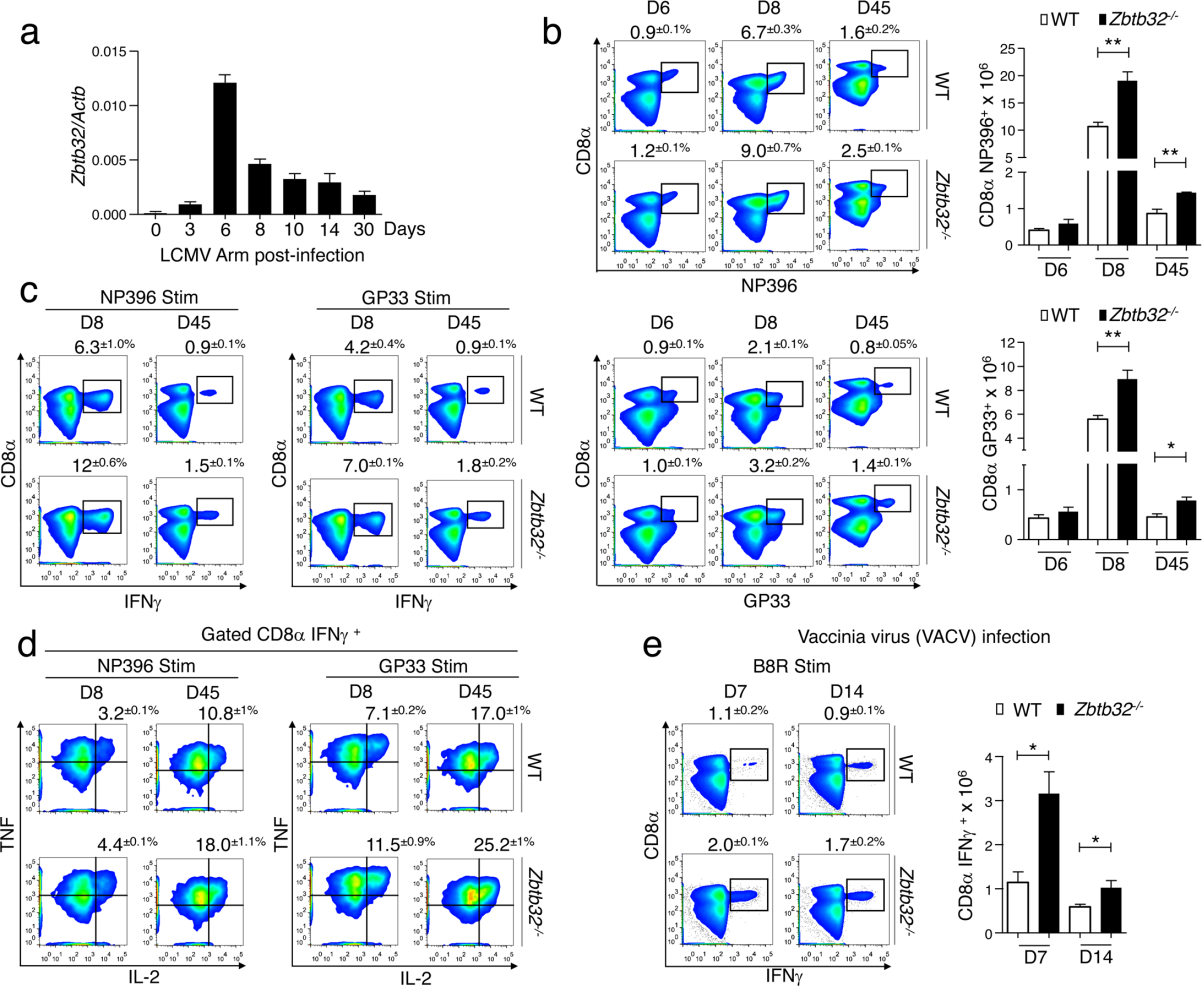
Enhanced CD8^+^ T cell response to acute virus infection in the absence of ZBTB32. (a) WT P14 CD8^+^ cells (5x10^4^) were transferred to recipients, which were then infected with LCMV-Armstrong. P14 cells were isolated and pooled from three mice at days 3, 6, 8, 10, 14 and 30 post-infection. *Zbtb32* mRNA was analyzed by quantitative RT-PCR relative to *Actb* mRNA. Day 0 represents P14 cells from naïve mice. The graph shows a compilation of data from three independent experiments; error bars represent the SEM. (b) The percentages ± SEM (left panels) and total numbers ± SEM (right graphs) of LCMV-specific CD8^+^ T cells in splenocytes from WT and *Zbtb32*^*-/-*^ mice were enumerated at days 6, 8 and 45 post-LCMV-Armstrong infection. Virus-specific CD8^+^ T cells were identified by Class-I MHC tetramer staining. Data are representative of two independent experiments for day 6 and three independent experiments for days 8 and 45, with 3 mice per genotype per experiment. (c,d) Splenocytes were isolated at days 8 and 45 from LCMV-infected WT and *Zbtb32*^*-/-*^ mice and were stimulated with NP396 (left) or GP33 (right) peptide followed by intracellular cytokine staining. Percentages ± SEM of (c) IFNγ ^+^ or (d) TNFα^+^ IL-2^+^ of IFNγ-producing virus-specific CD8^+^ T cells are depicted. Data are representative of three independent experiments with three mice per genotype per experiment. (e) Splenocytes from day 7 and 14 VACV-infected WT and *Zbtb32*^*-/-*^ mice were stimulated with VACV-specific B8R peptides followed by intracellular cytokine staining. Percentages ± SEM (left panels) or absolute numbers ± SEM (right graphs) of VACV-specific CD8^+^ T cells are depicted. Data are representative of two independent experiments with three mice per genotype per experiment.

To determine the function of ZBTB32 in CD8^+^ T cells, we first analyzed the kinetics of *Zbtb32* mRNA expression in CD8^+^ T cells responding to acute LCMV-Armstrong infection, and found a sharp peak of maximal expression at day 6 post-infection (Fig 2A). Next, we compared the CD8^+^ T cell responses of *Zbtb32*^*-/-*^ [21] versus wild type (WT) mice following LCMV-Armstrong infection. Uninfected *Zbtb32*^*-/-*^ mice showed normal development, with no apparent defects in the maturation or the proportions of lymphocytes in the thymus, spleen or lymph nodes (S2A and S2B Fig), as previously reported [20,21]. After infection, we found that *Zbtb32*^*-/-*^ mice had higher proportions and absolute numbers of LCMV-specific CD8^+^ T cells at days 8 and 45 post-infection (Fig 2B), a result confirmed by *ex vivo* IFNγ production (Fig 2C). Further, *Zbtb32*^*-/-*^ mice generated increased proportions of cells capable of producing IFNγ, TNFα and IL-2 simultaneously (Fig 2D), an indication of enhanced memory cell formation [27]. Viral titers in the spleens of infected *Zbtb32*^*-/-*^ mice were similar to WT controls, indicating that the increased magnitude of the CD8^+^ T cell response was not due to impaired viral clearance (S3A Fig). Additionally, we observed no differences in granzyme B expression between the two groups of mice (S3B Fig). To test whether these findings were generalizable to other infection models, we examined T cell responses to Vaccinia virus (VACV) infection. As shown, splenocytes from *Zbtb32*^*-/-*^ VACV-infected mice had a higher proportion and absolute number of IFNγ^+^ virus-specific CD8^+^ T cells as compared to WT controls (Fig 2E and S3C Fig). Overall, these results indicate that ZBTB32 is induced early and functions to limit effector T cell responses during acute virus infections.

### ZBTB32 intrinsically regulates effector CD8^+^ T cell responses and memory development

**Fig 3:**
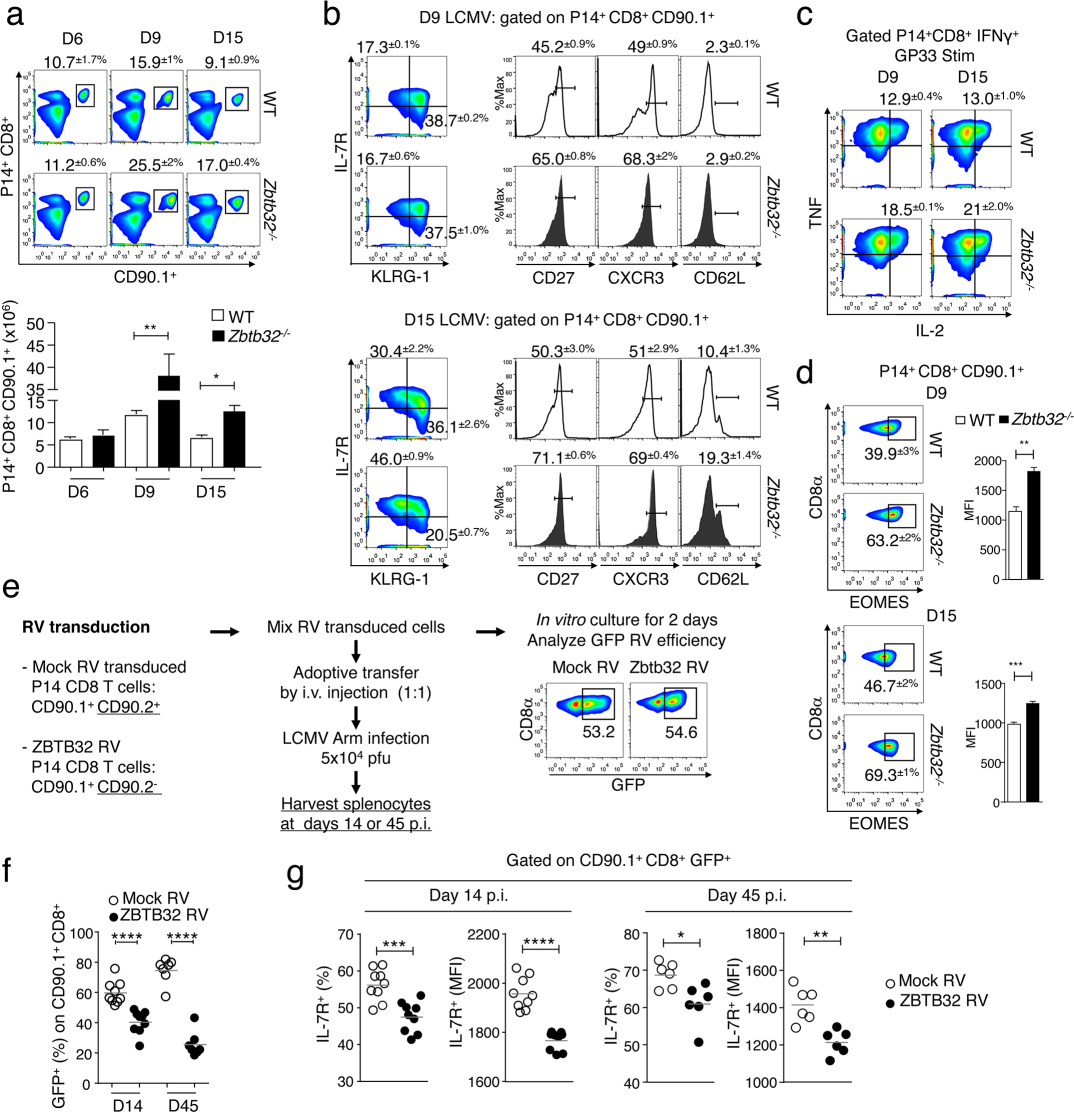
ZBTB32 is required intrinsically in CD8^+^ T cells to regulate memory cell development during acute virus infection. (a-b) WT or *Zbtb32*^*-/-*^ P14 splenocytes (CD90.1^+^) were adoptively transferred into CD90.2^+^ recipient mice followed by LCMV-Armstrong infection. (a) The percentages (top panels) and total numbers (graph below) of splenic P14 cells were enumerated at days 6, 9 and 15 post-infection. (b) At days 9 and 15, P14 cells were analyzed for memory markers as indicated. (c) P14 cells were cultured with GP33 peptide followed by intracellular cytokine staining. Plots show percentages ± SEM of TNF^+^ IL-2^+^ cells gated on P14 IFNγ^+^ cells. (d) Plots show percentages ± SEM of Eomes-positive splenic P14 cells and the MFI ± SEM of EOMES expression at day 9 (top) and day 15 (bottom) post infection. Data are representative of two independent experiments with three mice per genotype per experiment for day 6, and three independent experiments with three mice per genotype per experiment for days 9 and 15 post-infection. (e-g) Congenically-marked WT P14 cells stimulated *in vitro* with αCD3/CD28 for 24 hours were transduced with ZBTB32-expressing retrovirus (Zbtb32 RV) or mock retrovirus (mock RV) and then the two populations (mixed 1:1) were co-transferred into recipients, which were infected with LCMV-Armstrong. (e) A subset of transduced P14 cells was cultured *in vitro* for additional 2 days, and the transduction efficiency assessed by GFP fluorescence. At days 14 and 45 post-transfer and LCMV infection, P14 cells were analyzed for their (f) frequencies and for the (g) percentages ± SEM and MFI of IL-7R expression on each population. All data are compiled from two independent experiments with nine recipient mice for day 14 and six recipient mice for day 45.

Previously-reported data examined the responses of *Zbtb32*^*-/-*^ versus WT CD8^+^ T cells in mixed bone marrow chimeras infected with MCMV. In this study, MCMV-specific *Zbtb32*^*-/-*^ CD8^+^ T cells showed enhanced expansion compared to controls at D7 post-infection, indicating a CD8^+^ T cell-intrinsic role for ZBTB32 [24]. To address this issue more directly, *Zbtb32*^*-/-*^ and WT P14 splenocytes were transferred into WT recipients, followed by infection with LCMV-Armstrong. At days 9 and 15, we observed a higher proportion and absolute number of *Zbtb32*^*-/-*^ compared to WT P14 cells (Fig 3A), as well as a greater proportion of *Zbtb32*^*-/-*^ P14 cells expressing the memory markers CD27 and CXCR3 [28,29] at day 9 post-infection (Fig 3B, top). By day 15 post-infection, *Zbtb32*^*-/-*^ P14 cells were enriched in memory precursor effector cells (MPEC; IL-7R^hi^ KLRG-1^lo^) and a greater proportion expressed CD27, CXCR3 and CD62L compared to WT controls (Fig 3B, bottom). *Zbtb32*^*-/-*^ P14 cells were also enriched for triple IFNγ, TNFα, and IL-2 cytokine-producing populations (Fig 3C), and had increased expression of the transcription factor EOMES that promotes persistence of memory CD8^+^ T cells [30] (Fig 3D). These data indicated that ZBTB32 expression in CD8^+^ T cells limited effector T cell expansion and the generation of memory precursor cells.

Overall these findings suggested that persistent ZBTB32 expression would suppress T cell responses and memory generation. To test this, activated P14 cells transduced with retroviruses (RV) expressing ZBTB32 or a mock RV control were mixed 1:1, and co-transferred into recipient mice that were then infected with LCMV-Armstrong (Fig 3E). Expression of GFP after *in vitro* culture indicated that there were similar transduction efficiencies for each RV (Fig 3E). At days 14 and 45 post-infection, P14 cells transduced with the ZBTB32-RV were reduced in proportion compared to those transduced with the mock-RV control (Fig 3F). Furthermore, ZBTB32-RV transduced GFP^+^ cells at days 14 and 45 had a reduced proportion and MFI (mean fluorescent intensity) of IL-7R expression compared to control cells (Fig 3G). These gain-of-function studies confirmed that ZBTB32 normally functions to limit T cell responses and the generation of memory CD8^+^ T cells.

### Functional memory CD8^+^ T cell generation is enhanced in the absence of *Zbtb32*

**Fig 4:**
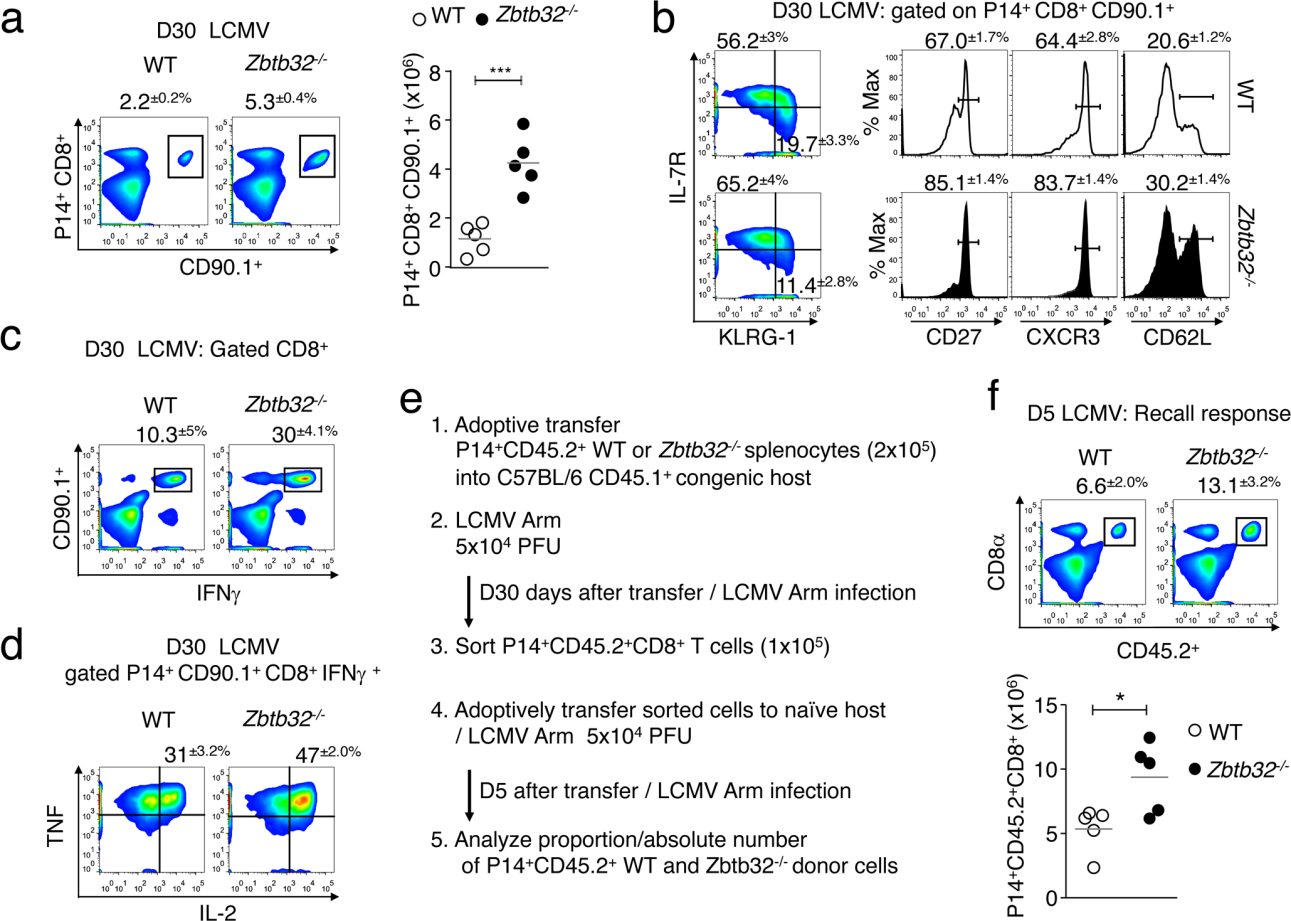
*Zbtb32*^*-/-*^ memory CD8^+^ T cells are functionally superior to WT memory cells. (a-d) WT or *Zbtb32*^*-/-*^ P14 splenocytes (CD90.1^+^) were adoptively transferred into CD90.2^+^ recipient mice followed by LCMV-Armstrong infection. (a) The percentages (left panels) and total numbers (graph at right) of splenic P14 cells were enumerated at day 30 post-infection. (b) At day 30, P14 cells were analyzed for memory markers as indicated. (c,d) P14 cells were cultured with GP33 peptide followed by intracellular cytokine staining. Plots show percentages ± SEM of TNF^+^ IL-2^+^ cells gated on P14 IFNγ^+^ cells. Data are representative of two independent experiments with three mice per genotype per experiment for day 30 post-infection. (e,f) At day 30 post-transfer and LCMV infection, WT or *Zbtb32*^*-/-*^ P14 cells were isolated, and 1x10^5^ of each population were transferred into naïve recipients, which were challenged with LCMV-Armstrong. At day 5 post-challenge, the percentages ± SEM (top) and numbers ± SEM (bottom) of P14 cells were analyzed in the spleen (f). Data are representative of two independent experiments with five mice per genotype of donor cells per experiment.

To address whether differences between WT and *Zbtb32*^*-/-*^ T cells were maintained into the memory phase, transferred *Zbtb32*^*-/-*^ and WT P14 cells were analyzed at day 30 post-LCMV-Armstrong infection. We found approximately three-fold more *Zbtb32*^*-/-*^ P14 than WT cells (Fig 4A). Furthermore, *Zbtb32*^*-/-*^ P14 cells were enriched in MPEC and had greater proportions of cells expressing CD27, CXCR3 and CD62L (Fig 4B), correlating with enhanced cytokine production (Fig 4C and 4D). To confirm that memory *Zbtb32*^*-/-*^ P14 cells were in fact *bona fide* memory T cell populations, we tested their recall response to a secondary LCMV challenge. *Zbtb32*^*-/-*^ or WT P14 cells were sorted at day 30 post-primary LCMV-Armstrong infection, and equal numbers were adoptively transferred into naïve hosts, which were then challenged with LCMV-Armstrong (Fig 4E). At day 5 post-challenge, a higher proportion and absolute number of *Zbtb32*^*-/-*^ P14 cells were found compared to WT controls (Fig 4F), indicating that, on a per-cell basis, *Zbtb32*^*-/-*^ CD8^+^ memory T cells were able to expand more robustly to secondary challenge compared to controls.

### Impaired survival and enhanced immunopathology following LCMV-clone 13 infection of *Zbtb32*^*-/-*^ mice

**Fig 5:**
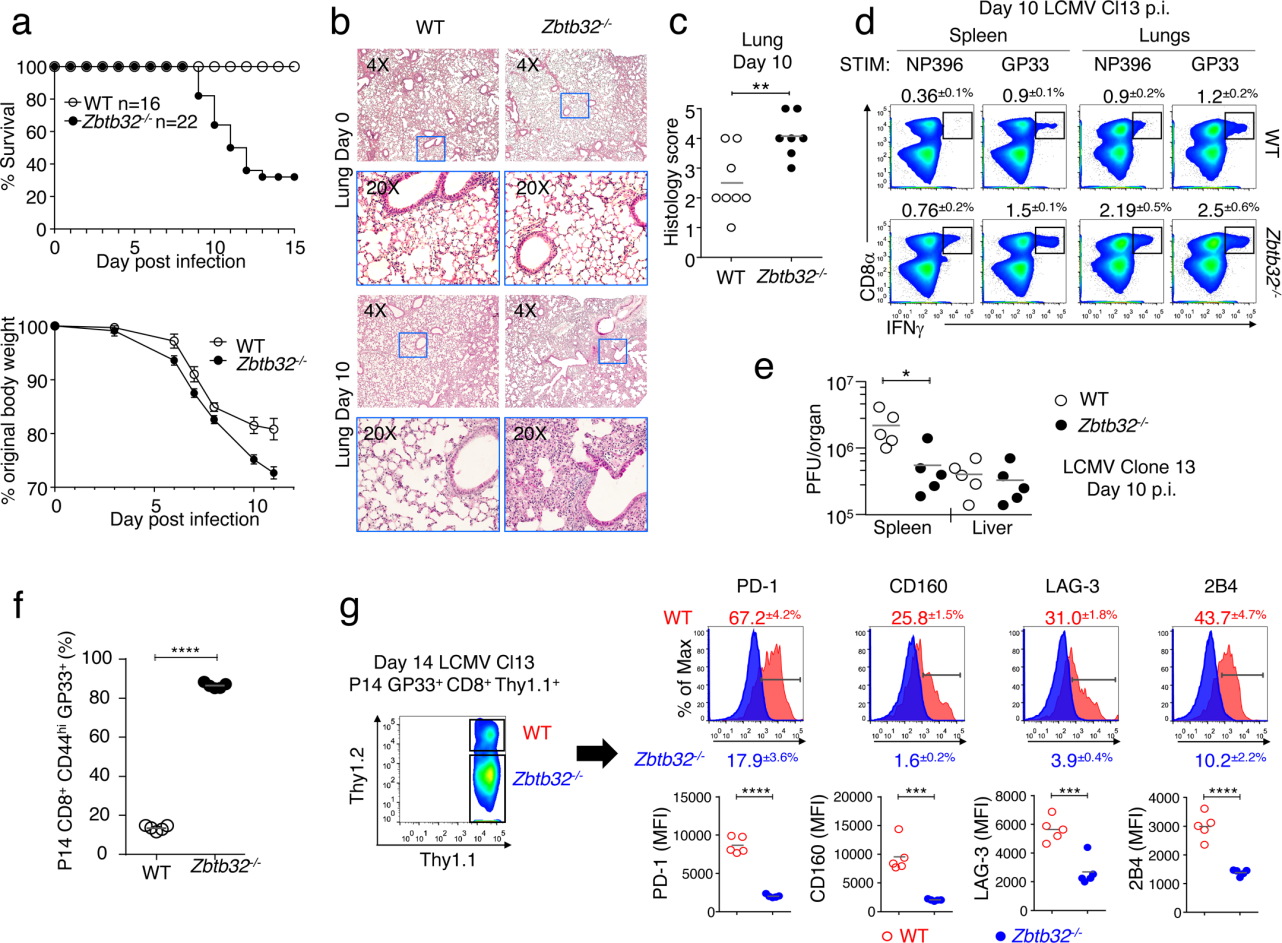
Increased immunopathology and reduced survival of *Zbtb32*^*-/-*^ mice in response to chronic LCMV infection. WT or *Zbtb32*^*-/-*^ mice were infected intravenously with high dose LCMV-clone 13. (a) The percent survival (top) and percentage of original body weight (bottom) of mice were recorded as indicated. The graphs include compilations of three independent experiments; error bars represent the SEM (bottom). (b,c) At days 0 and 10 post-infection, lung sections were stained with hematoxylin and eosin and displayed at 4x (top) or 20x (below) (b) and histology of lung sections at day 10 was scored (c) on a five-point scale (0–5) in a blind study. The criteria used for scoring include pulmonary oedema (pink material in air spaces), hemorrhage, necrotizing bronchiolitis (NB), interstitial mononuclear infiltration, and presence of bronchus-associated lymphoid tissue (BALT). Data are compilation of two independent experiments with eight mice for WT and seven for *Zbtb32*^-/-^. (d) At day 10 post-infection, lymphocytes from spleen and lungs were stimulated with NP396 or GP33 peptides followed by intracellular cytokine staining. The percentages ± SEM of IFNγ^+^ CD8^+^ T cells are depicted. Data are a representative of two independent experiments with three mice per genotype per experiment. (e) LCMV-clone 13 titers in spleen and liver were determined by plaque assay at day 10 post-infection. Data are representative of two independent experiments with five mice per genotype. (f,g) Congenically-marked WT and *Zbtb32*^*-/-*^ P14 cells mixed 1:1 were co-transferred into recipients, which were infected with LCMV-clone 13. At day 14, P14 donor cells from surviving recipients were analyzed for their frequencies (f) and analyzed for expression of exhaustion markers as indicated (g). Plots show percentages ± SEM (g, upper panels) and the MFI of exhaustion marker expression (g, lower graphs) as indicated. Data are compilations from two independent experiments with five surviving of eight recipient mice.

Since *Zbtb32*^*-/-*^ mice generated an enhanced CD8^+^ T cell response to acute LCMV-Armstrong infection, we addressed whether ZBTB32 plays a role in regulating T cell exhaustion during chronic infection with LCMV-Clone 13 (clone 13) [31,32]. When *Zbtb32*^*-/-*^ mice were infected with high-dose clone 13 (2x10^6^ pfu/mouse), approximately 70% of the mice succumbed by two weeks post-infection (Fig 5A, top), and showed more severe weight loss than controls (Fig 5A, bottom). A previous study showed that mortality during high dose LCMV-clone 13 infection is often associated with increased lung immunopathology [32]. Lung sections from naïve and day 10 clone 13-infected *Zbtb32*^*-/-*^ and WT mice were examined and scored on an arbitrary scale from 1–5 (1 = healthy, 5 = severe disease). Lungs of infected *Zbtb32*^*-/-*^ mice at day 10 had increased pathology as compared to controls (Fig 5B), along with lung histology scores indicative of enhanced disease (Fig 5C).

Both spleens and lungs of clone 13-infected *Zbtb32*^*-/-*^ mice had approximately two-fold more virus-specific CD8^+^ T cells at day 10 post-infection compared to controls (Fig 5D). In addition, viral clearance in the spleens of *Zbtb32*^*-/-*^ mice was enhanced compared to WT controls, although no significant differences were observed in the liver at this time point (Fig 5E). CD4^+^ T cell-depletion of *Zbtb32*^*-/-*^ mice and WT controls prior to infection with clone 13 did not alter the survival of *Zbtb32*^*-/-*^ mice, although it did lead to a less severe loss in body weight of both WT and *Zbtb32*^*-/-*^ clone 13-infected mice (S4A and S4B Fig). To address whether ZBTB32 had a CD8^+^ T cell-intrinsic role in regulating exhaustion along with limiting T cell expansion, we performed an adoptive transfer experiment. WT and *Zbtb32*^*-/-*^ P14 cells were mixed 1:1, transferred into recipients, and examined at day 14 post-infection with clone 13. Not only did *Zbtb32*^*-/-*^ P14 cells dominate the response relative to WT P14 cells (Fig 5F), but the *Zbtb32*^*-/-*^ P14 cells had reduced expression of characteristic exhaustion markers, PD-1, CD160, LAG-3 and 2B4 (Fig 5G). Thus, these results demonstrated that the suppression of antiviral CD8^+^ T cell responses mediated by ZBTB32 in WT cells is critical in controlling excessive effector T cell responses and promoting T cell exhaustion to prevent immunopathology.

### ZBTB32 promotes repressive chromatin modifications by recruiting HDAC1 and HDAC2 to target genes

**Fig 6:**
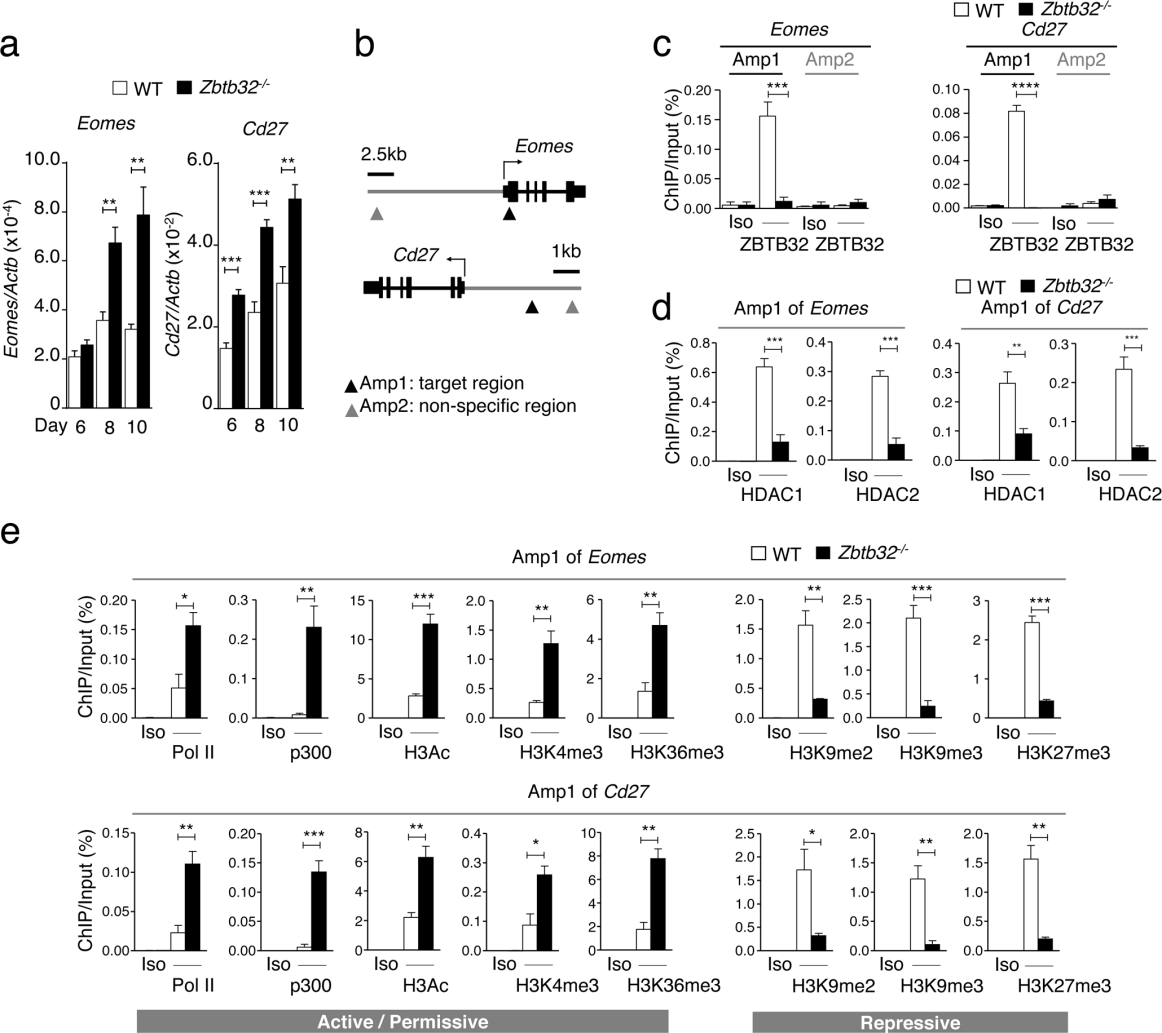
ZBTB32 represses *Eomes* and *Cd27* gene expression in CD8^+^ T cells by recruiting histone deacetylases 1 and 2. WT or *Zbtb32*^*-/-*^ P14 splenocytes were transferred into recipients, which were then infected with LCMV-Armstrong. At days 6, 8 and 10 post-infection, P14 cells were isolated and pooled from three mice per genotype for RNA isolation; chromatin was prepared at day 7 post-infection. (a) *Eomes* and *Cd27* mRNA levels were examined by quantitative RT-PCR relative to *Actb* mRNA. (b) Schematic of *Eomes* and *Cd27* gene loci showing position of specific (Amplicon 1; Amp1) and non-specific (Amplicon 2; Amp2) primers. In each case, Amp1 corresponds to putative ZBTB32 binding site. (c) The enrichment of ZBTB32 on *Eomes* and *Cd27* genes by chromatin immunoprecipitation (ChIP). (d) The enrichment of HDAC1 and HDAC2 on *Eomes* and *Cd27* genes by ChIP. (e) ChIP for Pol II, p300 or modified histone H3 at the *Eomes* and *Cd27* loci. Data are a compilation of three independent experiments; error bars represent the SEM. Iso; isotype control antibody.

To understand the mechanism by which ZBTB32 regulates immune response during LCMV-Armstrong infection, we chose to assess candidates genes previously shown to regulate effector-memory CD8^+^ T cell differentiation and survival [2–4,33], and compared mRNA levels in WT and *Zbtb32*^*-/-*^ P14 cells at several timepoints post-infection (Fig 6A and S5A Fig). Among these genes, mRNA and protein expression of *Eomes* and *CD27*, two genes known to promote the persistence and survival of memory CD8^+^ T cells [15,28–30], were significantly enhanced in the absence of ZBTB32 (Figs 6A, 3B and 3D). Therefore, we focused on understanding the mechanism by which ZBTB32 regulated expression of these two genes.

ChIP assays verified the direct regulation of *Eomes* and *CD27* by ZBTB32 (Amp1) (Fig 6B and 6C). As expected, there was no binding of ZBTB32 to any of these sites in *Zbtb32*^*-/-*^ CD8^+^ T cells, nor was ZBTB32 binding detected at non-specific regions (Amp2) of each gene (Fig 6C). Since ZBTB32 is known to recruit histone modifying enzymes to the *Il4* gene [19], we determined whether HDAC1 and HDAC2 were present at ZBTB32-binding regions of *Eomes* and *Cd27* in WT CD8^+^ T cells, and found both factors present; furthermore, this binding was greatly reduced in *Zbtb32*^*-/-*^ CD8^+^ T cells (Fig 6D). The ZBTB32-dependent binding of HDAC1 and HDAC2 was not detected at control loci (Amp2) of either gene (S6A Fig).

We next determined whether ZBTB32 regulated histone modifications and transcription of these genes. Along with high amounts of Pol II and p300 binding at Amp1 of *Eomes* and *Cd27*, CD8^+^ T cells isolated from *Zbtb32*^*-/-*^ mice at day 6 post-LCMV-Armstrong infection had increased amounts of H3Ac, H3K4me3 and H3K36me3, modifications that correlate with a permissive chromatin state [34], and reduced amounts of repressive H3K9me2, H3K9me3 and H3K27me3 modifications [34], compared to CD8^+^ T cells from WT mice (Fig 6E). In contrast, no ZBTB32-dependent differences in factor binding or repressive histone modifications were observed at non-specific regions (Amp2) of *Eomes* and *Cd27*, nor at the transcription start site (Amp) of *Cd8a* as a control (S6B–S6D Fig). Together, these data demonstrated that ZBTB32 induced a repressive chromatin state at regulatory regions of target genes in CD8^+^ T cells upon LCMV infection, thereby suppressing the transcription of genes important for the formation of long-lived memory T cells.

### ZBTB32 and Blimp-1 cooperate to bind to the *Eomes* and *Cd27* genes

**Fig 7:**
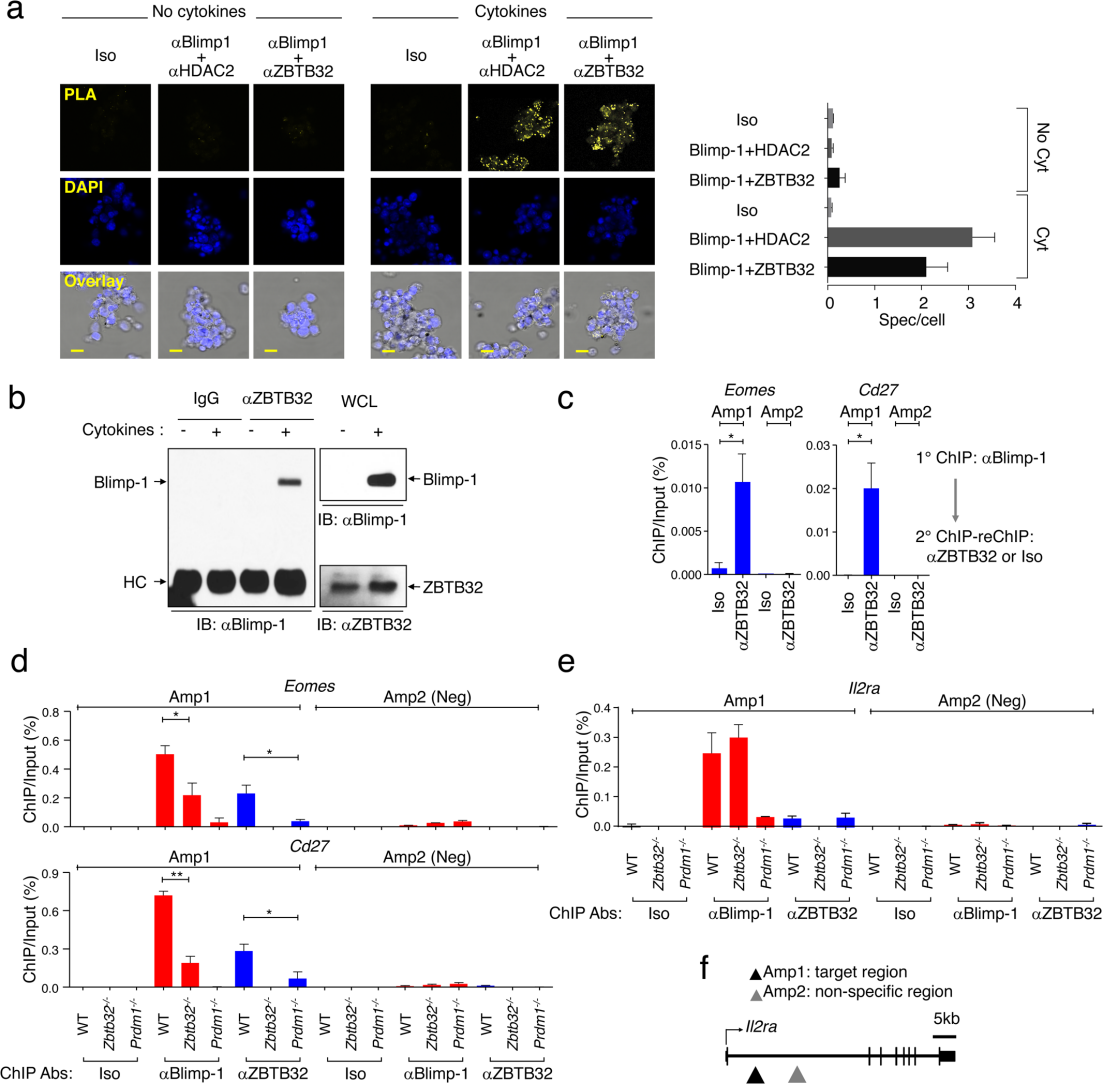
ZBTB32 and Blimp-1 bind cooperatively to target genes in activated CD8^+^ T cells. (a) WT CD8^+^ T cells pooled from three mice were stimulated with αCD3 and αCD28 for 2 days with or without a cocktail of cytokines containing IL-2, IL-4 and IL-12. Cells were harvested and stained with a mouse IgG and a rabbit IgG as negative controls (Iso), αBlimp-1 and αHDAC2, or αBlimp-1 and αZBTB32, followed by the Duolink proximity ligation assay. Samples were counter-stained for nuclei (blue; DAPI). Yellow signals demonstrate close proximity of the two proteins and data are representative of three independent experiments (left panels) and the graph (right panel) is a compilation of data from three independent experiments, and error bars represent the SEM. (b) WT CD8^+^ T cells pooled from three mice were stimulated with αCD3/CD28 for 2 days with or without a cocktail of cytokines containing IL-2, IL-4 and IL-12. Cells were harvested for immunoprecipitation with αZBTB32 or an IgG control antibody (left panel), followed by Immunoblotting (IB) with αBlimp-1. The presence of Blimp-1 and ZBTB32 in the whole cell lysates were confirmed (right panels). Data are representative of three independent experiments. WCL; whole cell lysate. (c) WT P14 cells were isolated from three recipient mice at day 7 post-LCMV-Armstrong infection, and chromatin was prepared. Primary ChIP assays were performed with antibodies to Blimp-1. Eluates from αBlimp-1 immunoprecipitates were re-precipitated with αZBTB32 or rabbit IgG (Iso) antibodies. Each ChIP eluate was amplified by Q-PCR for the indicated regions of the *Eomes* (left graph) and *Cd27* (right graph) genes. (d-f) WT, *Zbtb32*^*-/-*^ or *Prdm1*^*-/-*^ P14 cells were isolated from three recipient mice at day 7 post-LCMV-Armstrong infection, and chromatin was prepared. ChIP assays were performed with antibodies to Blimp1, ZBTB32 or mouse IgG plus rabbit IgG (Iso). ChIP eluates were amplified by Q-PCR for the indicated regions of *Eomes* (d, upper), *Cd27* (d, lower) and *Il2ra* (e). (f) Schematic of *Il2ra* gene loci. In each case amplicon 1 (Amp1) corresponds to putative Blimp-1 or ZBTB32 binding site and amplicon 2 (Amp2) to a negative control region. All graphs shown are from a compilation of three independent experiments and error bars represent the SEM.

ZBTB32 has been shown to bind to Blimp-1 in Raji B cell lines [22]. To address whether ZBTB32 interacted with Blimp-1 in activated CD8^+^ T cells, a single cell-based proximity ligation assay was performed (Fig 7A). Following TCR plus cytokine stimulation leading to high-level Blimp-1 [15] and ZBTB32 expression (Fig 1A), ZBTB32 and Blimp-1 were found to interact in CD8^+^ T cells (Fig 7A). As a positive control, we verified the interaction of Blimp-1 and HDAC2, as described previously (Fig 7A) [15]. The interaction of ZBTB32 and Blimp-1 was also confirmed by co-immunoprecipitation from activated primary T cells (Fig 7B). Furthermore, ZBTB32 and Blimp-1 bound together on target genes *Eomes* and *Cd27*, as revealed by sequential ChIP experiments (ChIP-reChIP) performed on chromatin from virus-specific T cells (Fig 7C). These data indicated that ZBTB32 is in close proximity to Blimp-1 on both the *Eomes* and *Cd27* genes, providing evidence that ZBTB32 and Blimp-1 may co-operatively regulate target genes in primary T cells activated *in vivo*.

*Cd27* [13,15] and *Eomes* [15] are Blimp-1 targets genes in CD8^+^ T cells. To determine if Blimp-1 and ZBTB32 bind cooperatively to shared targets, we examined the binding of each factor in virus-specific CD8^+^ T cells in the presence versus the absence of the other factor. The absence of *Prdm1* attenuated ZBTB32 binding to the regulatory regions of *Eomes* and *Cd27* at day 7 post-LCMV-Armstrong infection, and in reciprocal fashion, the *Zbtb32*-deficiency attenuated Blimp-1 binding (Amp1 in *Eomes* or *Cd27*) (Fig 7D). These data directly demonstrated that ZBTB32 and Blimp-1 co-operate in their binding to the regulatory regions of these two genes. We have previously reported that Blimp-1 regulates the *Il2ra* gene by direct binding on the regulatory region of its gene locus [15], as shown (Fig 7E); however, unlike *Eomes* and *Cd27*, Blimp-1 binding to this target gene was independent of ZBTB32. We conclude that a subset of Blimp-1-regulated genes requires the cooperative activity of ZBTB32, whereas other Blimp-1 target genes are ZBTB32-independent.

### The absence of both ZBTB32 and Blimp-1 enhances the generation of virus-specific CD8^+^ T cells

**Fig 8:**
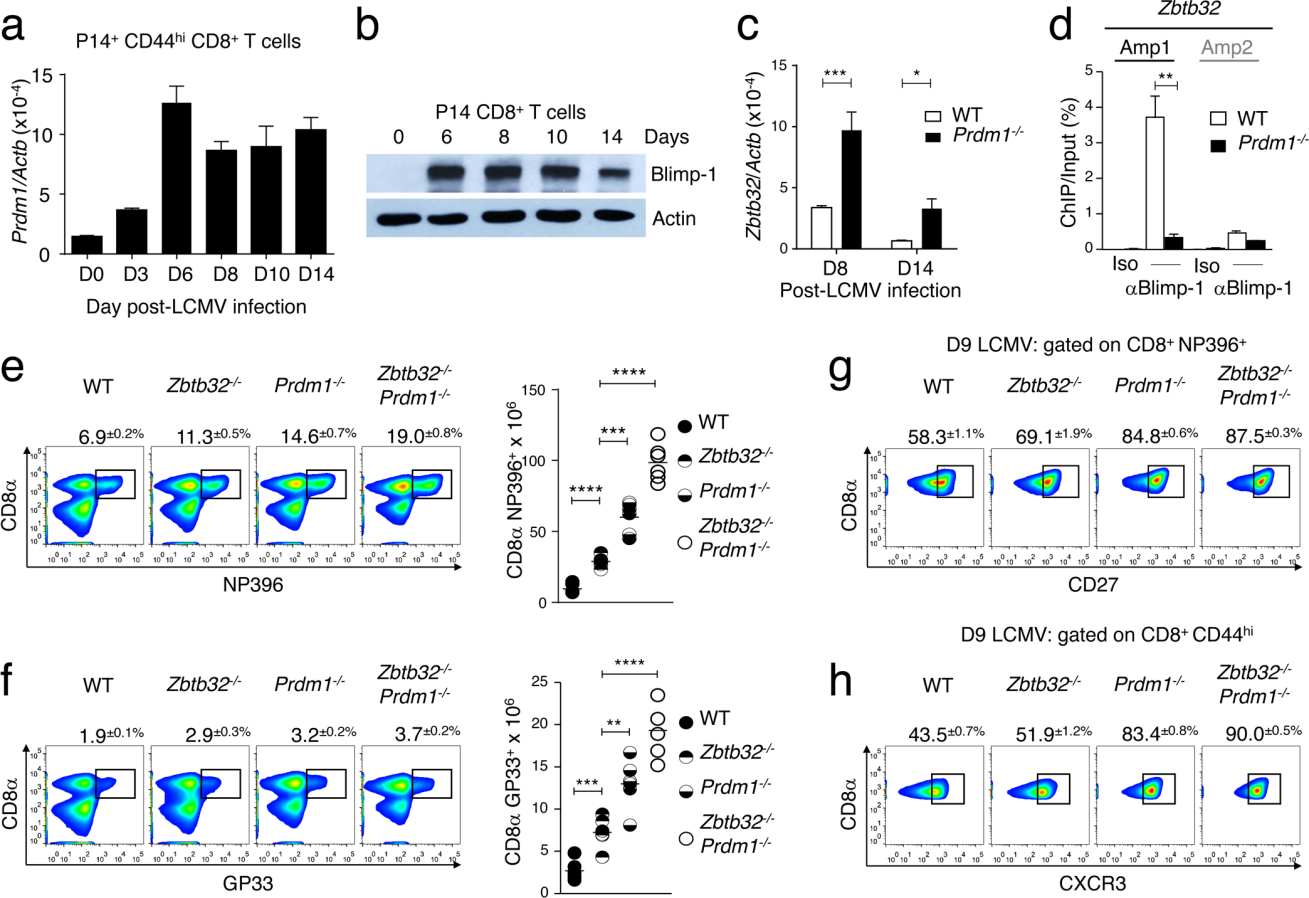
Non-redundant roles for ZBTB32 and Blimp-1 in anti-viral CD8^+^ T cell responses. (a,b) WT P14 cells were isolated from three recipient mice at the indicated days post-LCMV-Armstrong infection. (a) *Prdm1* mRNA was quantified. (b) Lysates were immunoblotted for Blimp-1 protein. Data are from three independent experiments; error bars represent SEM. (c,d) WT or *Prdm1*^*-/-*^ P14 cells were isolated from three recipient mice at days 8 and 14 post-LCMV-Armstrong infection. *Zbtb32* mRNA was quantified (c) and chromatin was prepared for ChIP assays with antibodies to Blimp1 or mouse IgG (Iso). ChIP eluates were amplified by Q-PCR for *Zbtb32* gene. In each case amplicon 1 (Amp1) corresponds to putative Blimp-1 binding site and amplicon 2 (Amp2) to a negative control region. All graphs shown are from a compilation of three independent experiments and error bars represent the SEM. (e-h) LCMV-specific CD8^+^ T cells in splenocytes from WT, *Zbtb32*^*-/-*^, *Prdm1*^*-/-*^ and *Zbtb32*^*-/-*^ *Prdm1*^*-/-*^ mice were analyzed at day 9 post-LCMV-Armstrong infection. Percentages ± SEM (left panels) and total numbers (graph at right) of LCMV-specific CD8^+^ T cells are depicted (e,f). LCMV-specific CD8^+^ T cells (g) and total CD8^+^CD44^hi^ T cells (h) were analyzed for memory markers as indicated. Data are representative of two independent experiments with three mice per genotype per experiment.

Blimp-1 is a transcriptional repressor known to regulate the differentiation of effector T cells [13–15,35,36]. Unlike the transient expression of ZBTB32 in CD8^+^ T cells responding to acute virus infection, Blimp-1 expression is maintained in virus-specific cells well into the memory time points [13,37]. Yet by day 14 post-LCMV infection, when Blimp-1 levels were still readily detectable (Fig 8A and 8B), CD8^+^ T cells have begun to express surface receptors characteristic of memory cells. This prompted us to investigate whether Blimp-1 regulated ZBTB32 expression, or vice-versa. We found that the transcript levels for *Zbtb32* were substantially elevated in *Prdm1*^*-/-*^ CD44^hi^ CD8^+^ T cells isolated at days 8 and 14 post-LCMV-Armstrong infection compared to WT controls (Fig 8C). In contrast, *Prdm1* transcript levels were slightly decreased at day 6 in the absence of ZBTB32, whereas no differences were observed at days 8 and 10 post-infection in the presence versus the absence ZBTB32 (S5A Fig). Consistent with the marked increase in *Zbtb32* mRNA levels in the absence of Blimp-1, ChIP experiments revealed that Blimp-1 bound to the regulatory region of the *Zbtb32* gene (Fig 8D and S5B Fig), indicating that Blimp-1 represses *Zbtb32* expression during the late stage of the anti-viral immune response.

The striking similarity between our data on anti-viral CD8^+^ T cell responses in *Zbtb32*^*-/-*^ mice compared to that seen following infection of *Prdm1*^*-/-*^ mice [13–15] prompted us to examine if the double-deficiency of ZBTB32 and Blimp-1 enhances the generation of virus-specific CD8^+^ T cells, compared to a single-deficiency of each factor. While each single knockout line had increased numbers of LCMV-specific CD8^+^ T cells, the double-deficient mice had a further increase, consistent with elevated numbers of virus-specific cells expressing the memory markers CD27 and CXCR3 (Fig 8E–8H). Interestingly, in spite of the aberrantly high expression of ZBTB32 in *Prdm1*^*-/-*^ T cells, ZBTB32 in the absence of Blimp-1 does not prevent the overexpansion of CD8^+^ effector and memory T cells. This may be due to the requirement for both factors to repress *Eomes* and *Cd27* expression. Overall, these results indicate that ZBTB32 functions together with Blimp-1 to limit both effector T cell responses and memory development during acute virus infections.

## Discussion

Our data show that ZBTB32 plays a unique non-redundant role in limiting T cell responses and memory generation during acute virus infection, and that this regulation is essential to prevent lethality in a model of persistent virus infection. ZBTB32 is induced by TCR plus inflammatory cytokine signaling. Further, in the absence of ZBTB32, Blimp-1 fails to bind to the proximal regulatory regions of *Eomes* and *Cd27*, and that these genes fail to undergo repressive chromatin modifications, leading to premature upregulation of memory cell genes that promote long-term cell survival (see S7 Fig). These findings highlight the importance of preventing the accumulation of excessive numbers of effector T cells at early timepoints post-infection, as a means of preventing T cell-mediated immunopathology.

While Blimp-1 is well known to regulate the terminal differentiation of several cell types, ZBTB32 has been less well-studied in immune cells. Our data indicate that Blimp-1 and ZBTB32 acting together function in CD8 regulatory networks recently described [2–4]. In the absence of Blimp-1, virus-specific CD8^+^ T cells are increased in number, but have reduced expression of cytolytic molecules, such as perforin and granzyme B [13,14,36]. In contrast, Zbtb32-deficient T cells have no defect in cytolytic molecules, but like the Blimp-1-deficient CD8^+^ T cells, have increased potential to generate long-lived memory cells. These data indicate that Blimp-1 regulates a distinct set of genes, in addition to the target genes shared with ZBTB32. Our previous studies identified *Il2ra*, *Cd27* and *Eomes* as representative genes regulated by Blimp-1 [15]. We now show that *Cd27* and *Eomes* are co-regulated by ZBTB32, whereas *Il2ra* is a target of Blimp-1 alone.

The findings from recent studies help provide insight into the functions of ZBTB32 and Blimp-1 [5–7,38,39]. Of particular interest, the studies of Lin et al indicate a sudden change in CD8^+^ T cell lineage determination after the 3^rd^-4^th^ cell division post-stimulation, a timepoint that coincides with ~day 3 post-infection [38]. At this time, activated CD8^+^ T cells that initially resemble memory precursor cells (TCF1^hi^) begin to generate daughter cells with divergent lineage potentials, seen as TCF1^hi^ versus TCF1^lo^. The findings indicate that TCF1^hi^ cells continue to divide and generate both subsets; in contrast, TCF1^lo^ cells, which acquire features of terminal effector cells, can only generate TCF1^lo^ daughter cells that are unable to contribute to the long-lived memory pool. Tying these data to our studies of Blimp-1 and ZBTB32, we have found that Blimp-1 is a direct transcriptional repressor of *Tcf7* (the gene encoding TCF1) in CD4^+^ T cells, a mechanism that contributes to the divergent differentiation of activated CD4^+^ T cells into T_H_1 versus T_FH_ lineages [40]. We also show here that *Tcf7* is a Blimp-1 target in CD8^+^ T cells isolated from LCMV-Armstrong-infected mice (S8A and S8B Fig) [15]. Together, these data suggest that Blimp-1 and ZBTB32 function to establish the balance of these two groups of CD8^+^ effector T cells.

Consistent with this model, Blimp-1 and ZBTB32 expression peak in parallel with the robust proliferative expansion of the short-lived effector response. Given the strong influence of inflammatory cytokines on the up-regulation of both *Prdm1* and *Zbtb32* mRNA levels, together with the known role of these cytokines in promoting a more robust effector response [8–12], it is likely that the most robustly-proliferating cells are expressing high levels of Blimp-1 and ZBTB32. This scenario is consistent with our findings that *Zbtb32*^*-/-*^ anti-viral CD8^+^ T cells are both increased in numbers and exhibit a pronounced increase in memory cell markers at the peak of the response. Also consistent are previous studies showing increased proliferation of *Zbtb32*^*-/-*^ T cells following stimulation *in vitro* [18,20], a feature likely attributed to enhanced cell survival. This model proposes that the cells with the highest Blimp-1 and ZBTB32 expression would likely die, due to the repression of genes required for long-term survival, and the remaining less differentiated effector cells would further down-regulate ZBTB32 expression due to Blimp-1-mediated repression. This final phase would allow the remaining cells to re-express the memory cell markers needed for long-term survival, thereby generating the normal small proportion of memory cells. Our findings of increased numbers of long-lived memory cells in *Zbtb32*^*-/-*^ mice indicate the obvious dysregulation of this response in the absence of ZBTB32-mediated repression.

One surprising finding in our study was that *Zbtb32*^*-/-*^ mice show a high mortality rate following infection with high-dose LCMV-clone 13. In WT mice, fatality from clone 13 infection is avoided due to a process of clonal exhaustion, in which the majority of virus-specific effector CD8^+^ T cells undergo cell death, and many of the remaining cells become functionally nonresponsive [31]. In *Zbtb32*^*-/-*^ mice, virus-specific CD8^+^ T cells are more numerous and less exhausted as indicated with reduced expression of exhaustion markers, correlating with a reduction in viral titers in the spleen at day 10 post-high-dose LCMV-clone 13 infection. Our current data do not indicate whether the unrestrained expansion of *Zbtb32*^*-/-*^ CD8^+^ T cells results from enhanced responses to persistent antigen or to inflammatory cytokines. While adoptive transfer experiments, as well as data from mixed bone marrow chimeras [24], confirm that the altered CD8^+^ T cell response is intrinsic to the loss of ZBTB32 in CD8^+^ T cells, other transcription factors may function in a regulatory network together with ZBTB32 to control exhaustion during chronic infection [41–46]. Thus, de-repression of ZBTB32-regulated genes might account for the failure of *Zbtb32*^*-/-*^ T cells to undergo exhaustion, leading to the fatal immunopathology observed in response to LCMV-clone 13. These findings, that *Zbtb32*^*-/-*^ CD8^+^ T cells are refractory to clonal exhaustion in the presence of persistent antigen stimulation, raises the interesting possibility that manipulation of ZBTB32 activity may be useful in the context of cancer immunotherapy.

Our data identify ZBTB32 as a necessary co-factor for Blimp-1-mediated regulation of key memory cell genes, *Eomes* and *Cd27* in CD8^+^ T cells. Furthermore, our LCMV infection studies suggest that this cooperation is required to program anti-viral CD8^+^ T cells for the terminal effector fate, leading ultimately to cell death, a program that appears essential for efficient clonal exhaustion. Unlike Blimp-1, ZBTB32 is only transiently expressed over the course of the anti-viral response. These findings leave open the possibility that Blimp-1 in the absence of ZBTB32 may function together with other factors, such as HOBIT protein (homolog of Blimp-1 in T cells) expressed in tissue-resident lymphocytes [47–49]. In future, continued biochemical, genetic, and molecular analysis of anti-viral CD8^+^ T cells at each phase of the response will be invaluable in resolving the multiple cell subsets contributing to the overall population of protective anti-viral T cells.

## Materials and methods

### Mice

C57BL/6J male mice were purchased from the Jackson Laboratory (Bar Harbor, ME). P14 transgenic mice were bred onto CD90.1 and CD45.1 C57BL/6 backgrounds to distinguish the transgenic cells from wild-type (WT) cells after adoptive transfer into C57BL/6 (CD90.2^+^ CD45.2^+^) mice. *Zbtb32*^*-/-*^ mice and *Prdm1*^*-/-*^ mice were kindly provided by I-Cheng Ho (Harvard Medical School, Brigham and Women's Hospital) and Alexander Tarakhovsky (Rockefeller University), respectively [15,21,50] and *Zbtb32*^*-/-*^ mice were crossed to P14 TCR transgenic CD90.1 or CD45.1 mice for adoptive transfer studies.

### Ethics statement

This study was carried out in strict accordance with the recommendations in the Guide for the Care and Use of Laboratory Animals of the U. S. National Institutes of Health. All animal experiments were approved by the Institutional Animal Care and Use Committee (IACUC) of the University of Massachusetts Medical School (UMMS) (Animal Welfare Assurance #A-1068-15). Mice were bred and housed in specific pathogen free conditions at the UMMS in accordance with the guidelines of the IACUC of UMMS and all efforts were made to minimize suffering of mice.

### Virus infections

LCMV, strain Armstrong, was propagated in baby hamster kidney (BHK)-21 cells obtained from the American Tissue Culture Collection (ATCC) as previously described [51]. Mice were inoculated intraperitoneally (i.p.) with 0.1 ml containing 5 x 10^4^ PFU (plaque forming units) of LCMV in PBS. The Clone 13 variant of LCMV was propagated in BHK-21 cells [52] and titrated by plaque assay on african green monkey kidney (Vero) cells (ATCC). Mice were infected intravenously (i.v.) with 2 × 10^6^ (high dose) PFU of LCMV, strain Clone 13. In some experiments, mice were inoculated i.p. with 1 × 10^6^ PFU of Vaccinia virus (VACV), strain Western Reserve.

### Antibodies, flow cytometry and intracellular cytokine staining

Anti-mouse CD8α (53–6.7), CD4 (RM4-5), CD45.2 (104), CD27 (LG.310), CD122 (TM.BETA-1), CD25 (PC61), CD62L (MEL-14), TCRβ (H57-597) and NK1.1 (PK136) antibodies were purchased from BD Pharmingen. Anti-mouse CD44 (IM7), CD45.1 (A20), CD90.1 (HIS51), CD90.2 (53–2.1), CXCR3 (CXCR3-173), KLRG-1 (2F1), CD127 (A7R34), PD-1 (J43), CD160 (ebioCNX46-3), LAG-3 (ebioC9B7W), 2B4 (ebio244F4), EOMES (Dan11mag), Granzyme B (NGZB) and TCRδ (ebioGL3) antibodies were purchased from eBiosciences. To stain samples for intracellular antigens, FOXP3/Transcription factor staining buffer kit (eBiosciences) was used. For intracellular cytokine assays, samples were incubated for 5 hours ex-vivo with 1μM LCMV-specific NP_396-404_ or GP_33-41_ peptide or 1μM VACV-specific B8R or K3L peptide in the presence of Golgi Plug (BD Biosciences). Cells were then permeabilized using the cytofix/cytoperm kit (BD Biosciences) followed by intracellular staining for IFNγ (XMG1.2; eBiosciences), IL-2 (JES65H4; BD Pharmigen) and TNF (MP6-XT22; Biolegend). Samples were analyzed on an LSRII flow cytometer (BD Biosciences), and data were further analyzed using FlowJo (Tree Star).

### H2D^b^ MHC tetramer staining

For identification of virus-specific CD8^+^ T cells, cells from infected mice were incubated with PE- or APC-conjugated H-2D^b^-NP_396–404_ or -GP_33-41_ tetramers for 1 hour at 4°C followed by staining for surface antigens.

### Adoptive transfer studies, T cell isolation and CD4^+^ T cell depletion

Unless otherwise noted virus-specific CD8^+^ T cell responses were tracked by transferring 2x10^5^ (CD90.1^+^ or CD45.1^+^) splenocytes into congenic C57BL/6 hosts (CD90.2^+^ CD45.2^+^). Recall responses of *Zbtb32*^*-/-*^ or WT P14 CD8^+^ T cells at day 30 post-LCMV infection were compared by transferring sorted 1x10^5^ P14 CD8^+^ T cells (WT or *Zbtb32*^*-/-*^) into naïve hosts, which were subsequently infected with LCMV-Armstrong. Wherever noted, total CD8^+^ T cells were isolated with the CD8^+^ T Cell Isolation Kit II (Miltenyi Biotec), and LCMV-specific P14^+^ CD8^+^ T cells were further sorted on a FACS Aria cell sorter (BD Biosciences). WT and *Zbtb32*^*-/-*^ mice were depleted of CD4^+^ T cells by i.p. injection of anti-CD4 (clone GK1.5) or control IgG2b (Iso) at a dose of 1mg/mouse at day -1 and day 3 of LCMV-clone 13 infection.

### Plaque assays for LCMV titers

Spleens or livers from LCMV-infected WT, *Prdm1*^*-/-*^ or *Zbtb32*^*-/-*^ mice were homogenized and virus was tittered by plaque assay on Vero cells, as previously described [51].

### Histology

Lung tissue sections from high dose LCMV-Clone 13-infected mice were fixed in 10% paraformaldehyde and stained with hematoxylin and eosin (H&E).

### Quantitative RT-PCR

Total RNA was isolated, converted to cDNA, and analyzed by real-time quantitative PCR amplification on a Bio-Rad iCycler (Bio-Rad), using the iQ SYBR Green Supermix (Bio-Rad) as previously described [15]. The primers used for RT-PCR were described in S9 Fig. The primers for *Cd27* and *Actb* were purchased from Real Time Primers, LLC.

### Proximity ligation assay (PLA) and confocal microscopy

CD8^+^ T cells were cyto-spun onto positively-charged microscope slides (Fisher, 12-550-20) and washed with cold PBS twice, followed by fixation with 4% paraformaldehyde at 25°C for 10 min. Fixed cells were washed with PBS twice and permeabilized in 0.5% Triton X-100/PBS at 4°C for 6 min, followed by washing with 70% EtOH. After blocking samples in the Duolink II blocking solution for 30 min at 37°C, samples were incubated at 4°C overnight with αBlimp-1 and αHDAC2, αBlimp-1 and αZBTB32, or mouse IgG and rabbit IgG as a control, followed by the Duolink II proximity ligation assay according to the manufacturer’s instructions. Samples were counter-stained for nuclei (blue; DAPI). The signals (Red) from each pair of PLA probes were detected using laser-scanning confocal microscopy (Leica TCS SP5 II) with a 63x phase contrast oil immersion objective (numerical aperture = 1.3). The nuclei images were captured using the UV laser. Duolink II Detection kit (DUO92008), Duolink II PLA probe Mouse Plus (DUO92001) and Duolink II PLA probe Rabbit Minus (DUO92005) were purchased from Olink Bioscience.

### Retrovirus production and transduction

For production of retroviruses, human embryonic kidney 293T cells obtained from the American Tissue Culture Collection (ATCC) were transfected with 2 μg of retroviral DNA (Zbtb32 in pMigR1-GFP or pMigR1-GFP empty vector) and 1μg of pCL-Eco packaging DNA (Addgene), and retroviral supernatants were collected after two days. The *Zbtb32* cDNA was kindly provided by I-Cheng Ho (Harvard Medical School, Brigham and Women's Hospital) and subcloned into pMigR1-GFP vector, and then verified by DNA sequencing. Congenically-marked WT P14 CD8^+^ T cells were stimulated *in vitro* with αCD3 and αCD28 (eBiosciences) for 24 hours, and then transduced by spin-infection (2000 rpm, 25°C, 1 hour) with ZBTB32-expressing retrovirus (Zbtb32 RV) or mock retrovirus (mock RV). The two populations were mixed 1:1, and then 1x10^6^ cells were transferred into recipients, which were infected with LCMV-Armstrong. A subset of transduced P14 cells was cultured *in vitro* for an additional 2 days, and the transduction efficiency assessed by GFP fluorescence. At days 14 and 45 post-transfer and -LCMV infection, P14 cells were analyzed for their frequencies and for IL-7R expression on each population.

### Chromatin immunoprecipitation assay (ChIP) and sequential chromatin immunoprecipitation assays (ChIP-reChIP)

CD8^+^ T cells were fixed for 10 min at 25°C with 1% formaldehyde, and then quenched for 5 min at 25°C with 125mM Glycine (Sigma-Aldrich). The cells were washed twice in ice-cold 0.5% BSA-PBS. ChIP analysis was performed on 2x10^5^ cells using the ChIP Assay Kit (Millipore) following the manufacturer’s instructions. The antibodies used were αSTAT1 (Santa Cruz, sc-346), αSTAT4 (Santa Cruz, sc-486), αSTAT5 (R&D systems, PA-ST5A), αSTAT6 (Santa Cruz, sc-374021), αBlimp-1 (Santa Cruz, sc-66015), αHDAC1 (Abcam, ab7028-50), αHDAC2 (Invitrogen, 51–5100), αPol II (Santa Cruz, sc-9001), αp300 (Santa Cruz, sc-584), αH3Ac (Millipore, 06–599), αH3K4me3 (Millipore, 17–614), αH3K36me3 (Abcam, ab9050), αH3K9me2 (Abcam, ab-1220), αH3K9me3 (Millipore, 17–625), or αH3K27me3 (Abcam, ab-6002). The αZBTB32 antibody was generated by NeoBiolab, by immunizing rabbits with the chemically synthesized peptide: cys-GLGSPGEKQKPEKDFRSN (amino acids 141–160) as previously described [19]. ZBTB32-specific antibodies were affinity purified by binding to beads conjugated with a GST-fusion protein containing the N-terminal 1–165 acids of ZBTB32 and then purified antibodies were verified using ChIP assay (S10 Fig). To identify potential ZBTB32 binding sites in the *Eomes*, *Cd27* and *Il2ra* gene loci, we used the reported ZBTB32 binding motif [19,53,54] and performed a motif search using the Motif Alignment and Search Tool in the MEME Suite (v4.12.0.). Immunoprecipitated DNA (2 μl from a total of 50 μl) was quantified by real-time quantitative PCR amplification on a Bio-Rad iCycler, using the iQ SYBR Green Supermix (Bio-Rad). As a control, input DNA purified from chromatin before immunoprecipitation was used.

For the second round of ChIP (ChIP-reChIP), eluates from αBlimp-1 or mouse IgG immunoprecipitation were taken prior to reverse-crosslinking, and were diluted 10-fold in ChIP dilution buffer, and diluted eluates were incubated at 4°C on rotator overnight with αZBTB32 or rabbit IgG coupled with Dynabeads Sheep-αRabbit I magnetic beads (Invitrogen, Dynabeads M-280 Sheep αRabbit IgG). Immune complexes with magnetic beads were collected on the magnet, washed 5 times with LiCl wash buffer (Millipore) and then washed two times with TE buffer. Immune complexes were eluted in 500μl elution buffer (1%SDS, 0.1M NaHCO_3_), followed by reverse-crosslinking in a 65°C water bath overnight. DNA fragments were recovered by a Qiagen PCR Cleanup kit. Real-time quantitative PCR amplification was performed with 5 μl from a total of 50 μl of the immunoprecipitated DNA. PCR primers for RT-PCR and for ChIP Q-PCR are described in S9 Fig.

### Statistical analysis

Statistical differences between samples were analyzed with an unpaired Students t test (*p≤0.05, **p≤0.01, ***p≤0.001, ****p≤0.0001). All error bars in the manuscript represent the Standard Error of the Mean (SEM).

## Supporting information

S1 Fig Inflammatory cytokines induce STAT binding and permissive chromatin modifications at regulatory regions of *Zbtb32*. (PDF)

S2 Fig Characterization of immune subsets in uninfected *Zbtb32*^*-/-*^ mice. (PDF)

S3 Fig Characterization of anti-viral immune responses in *Zbtb32*^*-/-*^ mice. (PDF)

S4 Fig Enhanced immuno-pathology in *Zbtb32*^*-/-*^ mice following LCMV-clone 13 infection is not dependent on CD4^+^ T cells. (PDF)

S5 Fig Expression of a selected set of signature genes in effector-memory CD8^+^ T cell differentiation. (PDF)

S6 Fig Chromatin immunoprecipitation data from control regions of *Eomes*, *Cd27* and *CD8a* genes. (PDF)

S7 Fig Model for CD8^+^ effector versus memory T cell regulation by ZBTB32 and Blimp-1. (PDF)

S8 Fig Blimp-1 regulates TCF1 expression in CD8^+^ T cells. (PDF)

S9 Fig Primers and ChIP Amplicon primers for PCR and quantitative PCR. (PDF)

S10 Fig Titration of ZBTB32 antibody. (PDF)
