# Supplementary material for: Transient expression of ZBTB32 in anti-viral CD8+ T cells limits the magnitude of the effector response and the generation of memory
Source: PLoS Pathog. 2017 Aug 21;13(8):e1006544. doi: 10.1371/journal.ppat.1006544 (PMC5578684; doi:10.1371/journal.ppat.1006544)
Supplement: S1 Fig — (PDF) [file ppat.1006544.s001.pdf]

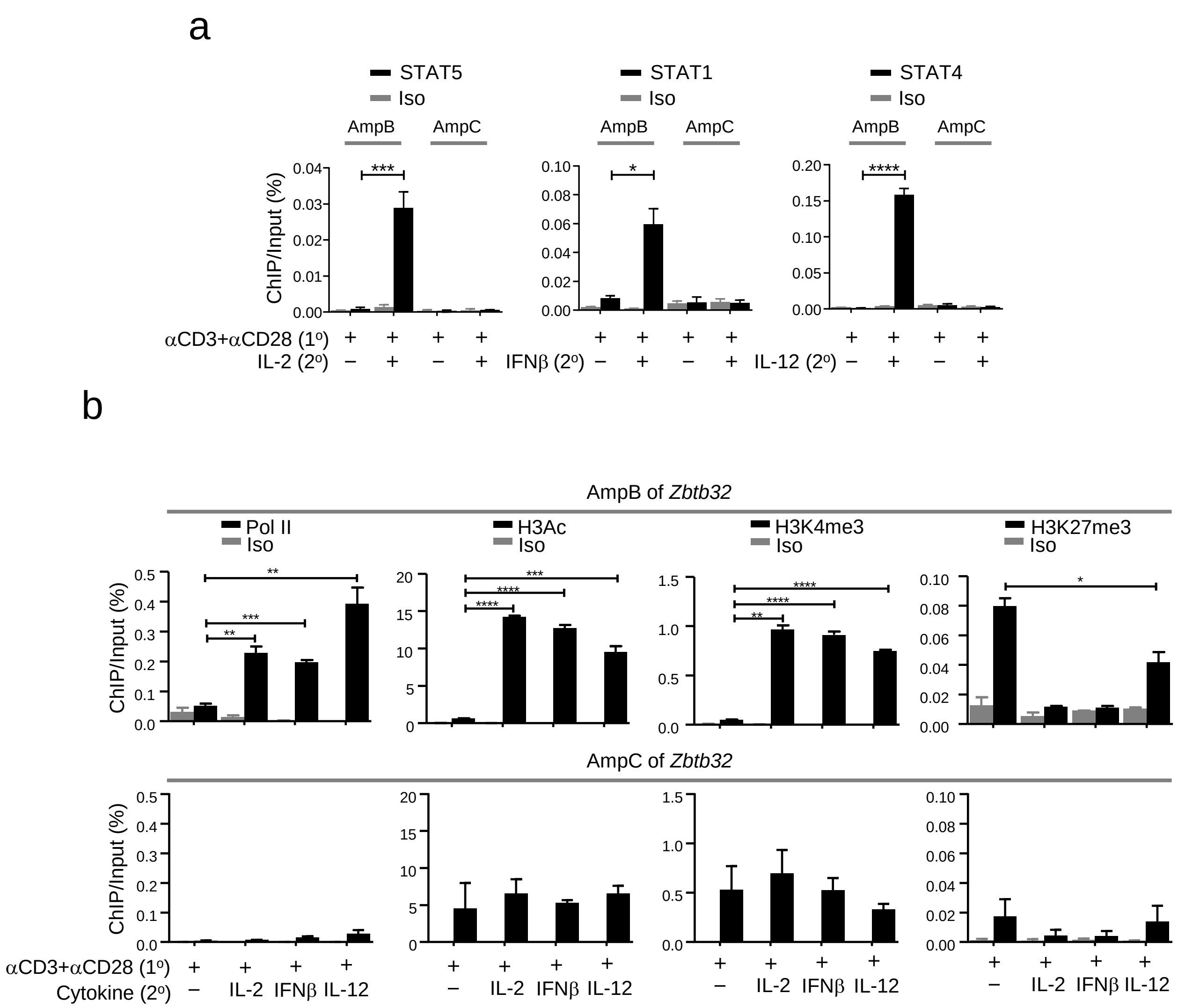

S1 Fig. Inflammatory cytokines induce STAT binding and permissive chromatin modifications at regulatory regions of *Zbtb32*.

(a) Pre-activated (1º) WT CD8+ T cells were stimulated with or without (2º) IL-2 (left), IFN-β (middle) or IL-12 (right) for 30 min and then analyzed by ChIP. Graphs show the binding of STAT-5 (left), STAT-1 (middle) or STAT-4 (right) at AmpB (target region) and AmpC (control region) of *Zbtb32*.

(b) Pre-activated (1º) WT CD8+ T cells were stimulated with or without (2º) IL-2 (left), IFN-β (middle) or IL-12 (right) for 3 hours. Chromatin was prepared for Pol II, H3Ac, H3K3me3 and H3K27me3 ChIP analysis compared to isotype control (Iso). ChIP eluates were amplified by Q-PCR for the indicated regions of the *Zbtb32* gene locus (AmpB) and control (AmpC).

Data are compilations from three independent experiments; error bars represent SEM.
