# Supplementary material for: Transient expression of ZBTB32 in anti-viral CD8+ T cells limits the magnitude of the effector response and the generation of memory
Source: PLoS Pathog. 2017 Aug 21;13(8):e1006544. doi: 10.1371/journal.ppat.1006544 (PMC5578684; doi:10.1371/journal.ppat.1006544)
Supplement: S2 Fig — (PDF) [file ppat.1006544.s002.pdf]

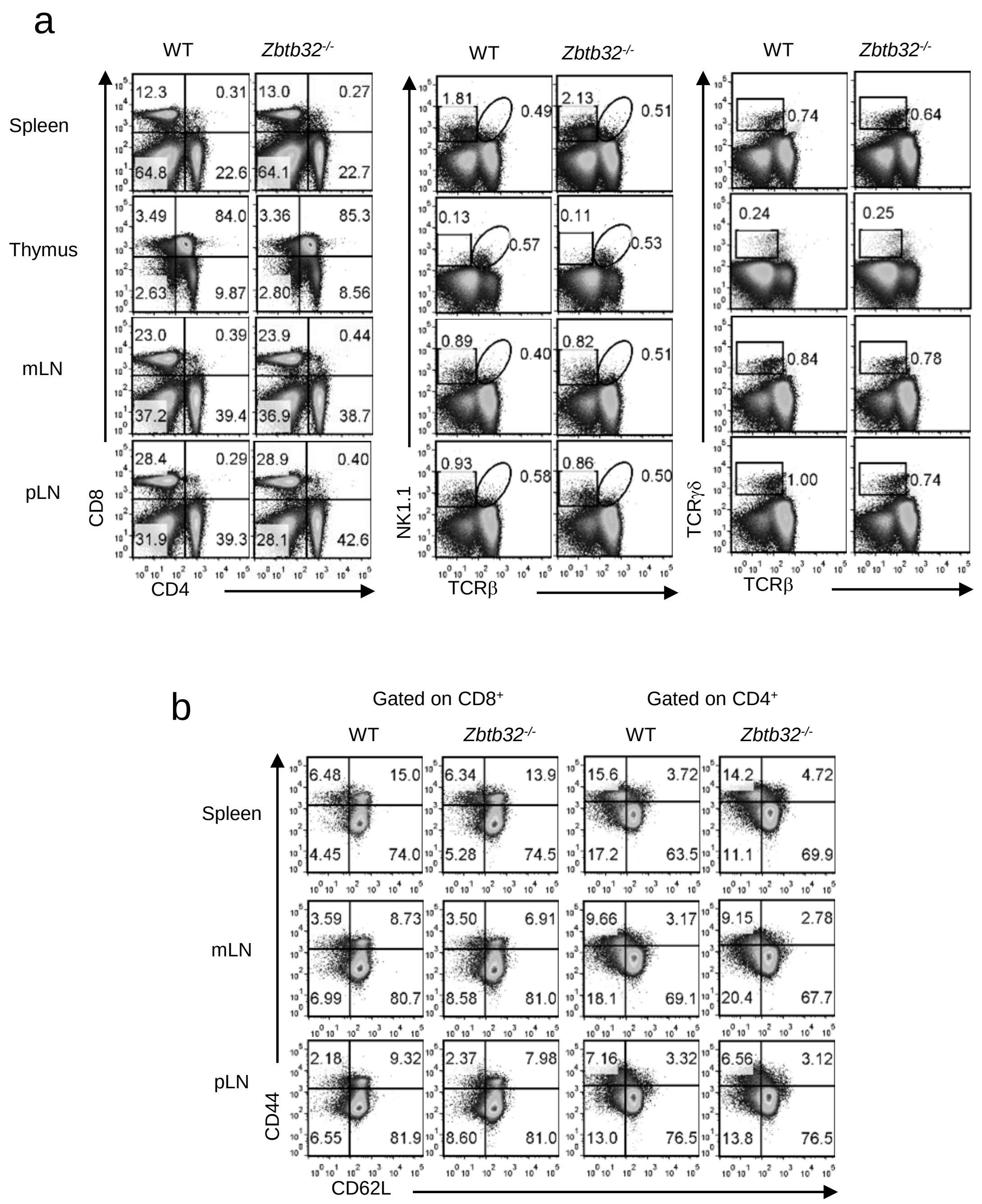

S2 Fig. Characterization of immune subsets in uninfected *Zbtb32*$^{-/-}$ mice.

(a) Spleen, thymus, mesenteric lymph nodes (mLN) and peripheral lymph nodes (pLN) from WT and *Zbtb32*$^{-/-}$ mice were analyzed by flow cytometry to characterize T cell subsets. Representative data are shown.

(b) The proportions of naive (CD44^{lo} CD62L^{hi}), effector (CD44^{hi}, CD62L^{lo}) and memory (CD44^{hi} CD62L^{hi}) CD8^{+} and CD4^{+} T cell subsets in spleen, mesenteric lymph nodes (mLN) and peripheral lymph nodes (pLN) of representative WT and *Zbtb32*$^{-/-}$ mice are shown.
