# Supplementary material for: Transient expression of ZBTB32 in anti-viral CD8+ T cells limits the magnitude of the effector response and the generation of memory
Source: PLoS Pathog. 2017 Aug 21;13(8):e1006544. doi: 10.1371/journal.ppat.1006544 (PMC5578684; doi:10.1371/journal.ppat.1006544)
Supplement: S3 Fig — (PDF) [file ppat.1006544.s003.pdf]

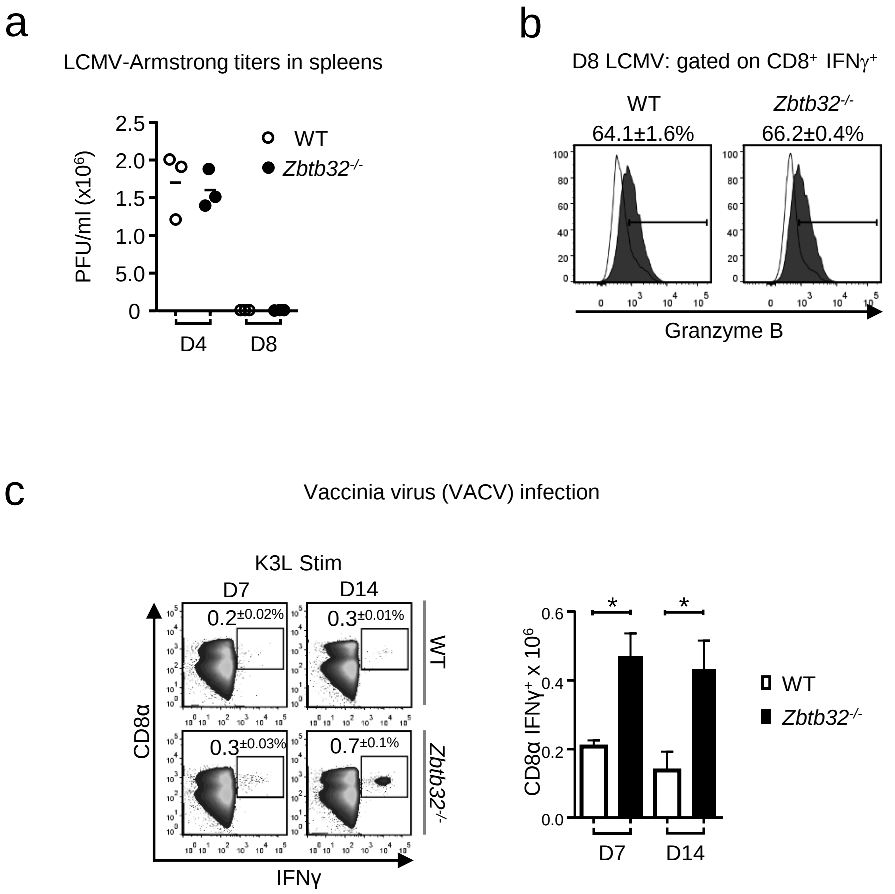

S3 Fig. Characterization of anti-viral immune responses in *Zbtb32*$^{-/-}$ mice.

(a) LCMV-Armstrong titers in spleens of infected WT and *Zbtb32*$^{-/-}$ mice were determined by plaque assay at days 4 and 8 post-infection. Data are representative of two independent experiments.

(b) Splenocytes were isolated at day 8 from LCMV-infected WT and Zbtb32-/- mice and were stimulated with GP33 peptide followed by intracellular cytokine staining. Percentages ± SEM of granzyme B+ of IFNγ-producing virus-specific CD8+ T cells are depicted. The open graphs show a negative control of grazyme B on IFNγ-non-producing cells. Data are representative of three independent experiments with three mice per genotype per experiment.

(c) Splenocytes from day 7 and 14 VACV-infected WT and *Zbtb32*$^{-/-}$ mice were stimulated with VACV-specific K3L peptides followed by intracellular cytokine staining. Percentages ± SEM (left panels) or absolute numbers ± SEM (right graphs) of VACV-specific CD8^{+} T cells are depicted. Data are representative of two independent experiments with three mice per genotype per experiment.
