# Supplementary material for: Transient expression of ZBTB32 in anti-viral CD8+ T cells limits the magnitude of the effector response and the generation of memory
Source: PLoS Pathog. 2017 Aug 21;13(8):e1006544. doi: 10.1371/journal.ppat.1006544 (PMC5578684; doi:10.1371/journal.ppat.1006544)
Supplement: S4 Fig — (PDF) [file ppat.1006544.s004.pdf]

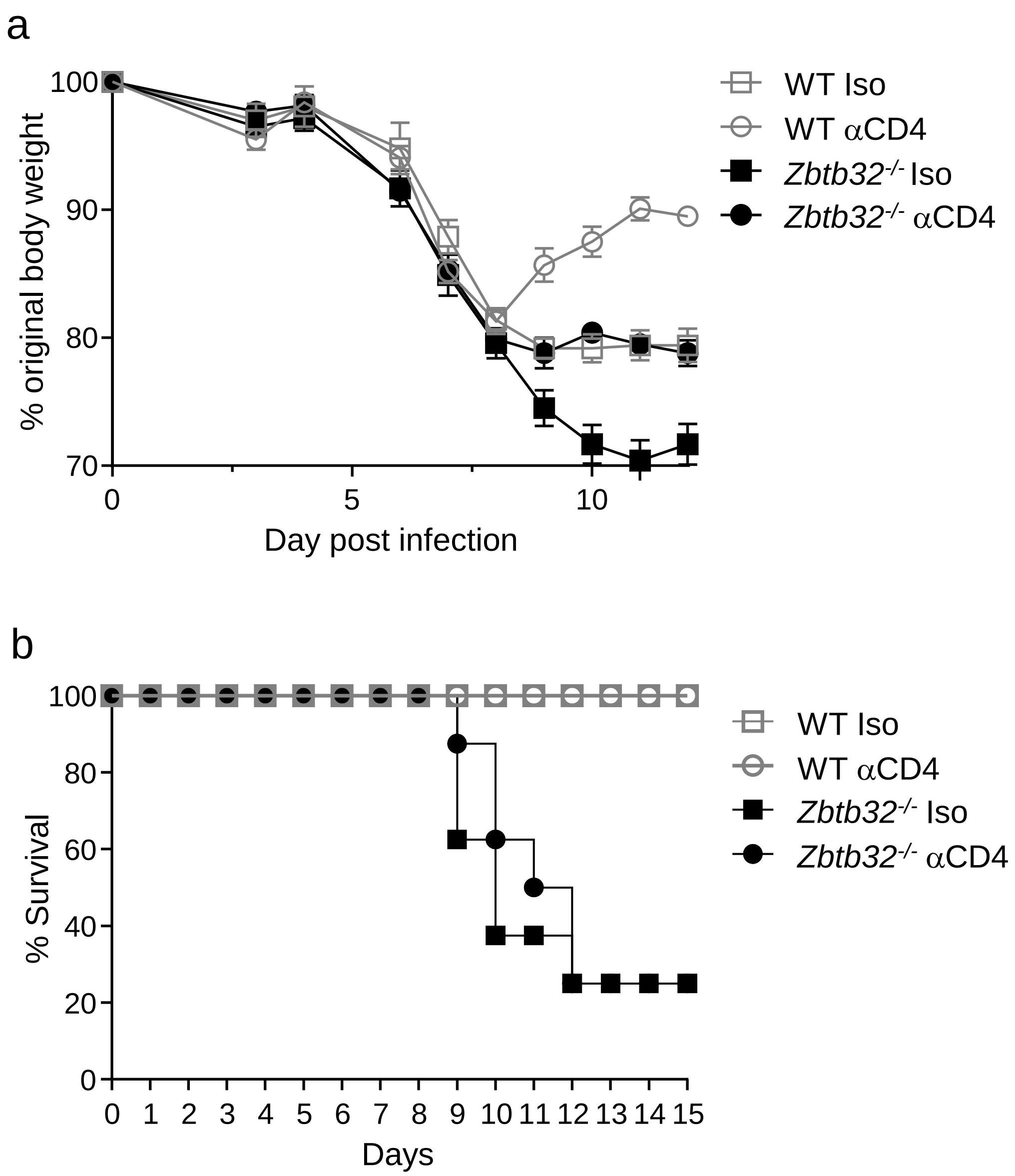

S4 Fig. Enhanced immuno-pathology in *Zbtb32$^{-/-}$* mice following LCMV-clone 13 infection is not dependent on CD4^{+} T cells.

(a,b) WT and *Zbtb32$^{-/-}$* mice were depleted of CD4^{+} T cells by i.p. injection of either anti-CD4 (αCD4) or control IgG2b (Iso) at day -1 and day 3 of LCMV-clone 13 infection. The percentage of original body weight ± SEM (a) and percent survival (b) of mice were recorded as indicated. Data were generated with 6 mice for WT Iso, 4 mice for WT αCD4, 8 mice for *Zbtb32$^{-/-}$* Iso and 8 mice for *Zbtb32$^{-/-}$* αCD4 conditions.
