# Supplementary material for: Transient expression of ZBTB32 in anti-viral CD8+ T cells limits the magnitude of the effector response and the generation of memory
Source: PLoS Pathog. 2017 Aug 21;13(8):e1006544. doi: 10.1371/journal.ppat.1006544 (PMC5578684; doi:10.1371/journal.ppat.1006544)
Supplement: S5 Fig — (PDF) [file ppat.1006544.s005.pdf]

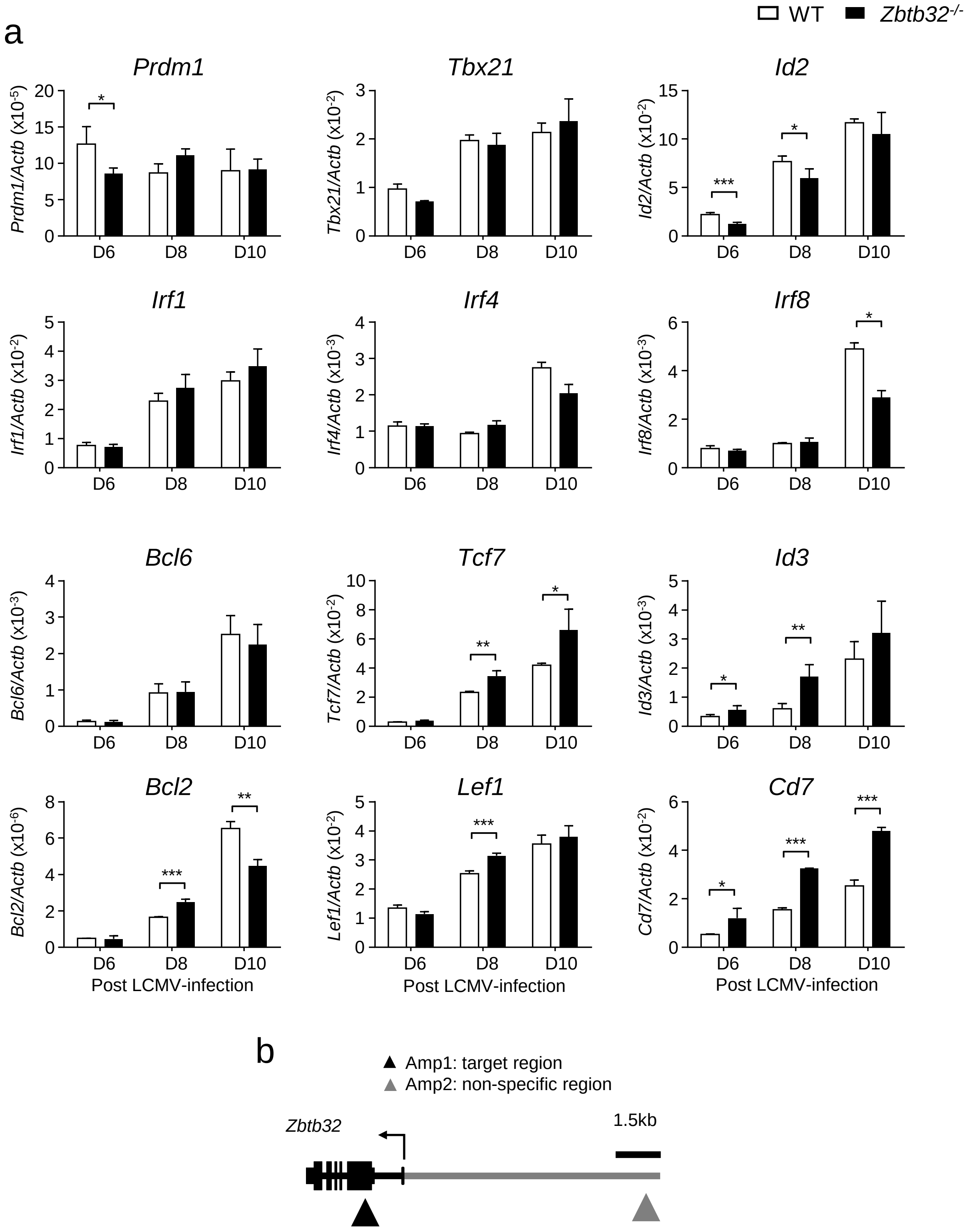

S5 Fig. Expression of a selected set of signature genes in effector-memory CD8^{+} T cell differentiation.

(a) WT or *Zbtb32*$^{-/-}$ P14 splenocytes were transferred into recipients, which were then infected with LCMV-Armstrong. At days 6, 8 and 14 post-infection, P14 cells were isolated and pooled from three mice per genotype for RNA isolation. mRNA levels of the indicated genes were examined by quantitative RT-PCR relative to *Actb* mRNA.

(b) Schematic of the *Zbtb32* gene locus. Amplicon 1(Amp1) corresponds to predicted Blimp-1 binding site and amplicon 2 (Amp2) to a negative control region.
