# Supplementary material for: Transient expression of ZBTB32 in anti-viral CD8+ T cells limits the magnitude of the effector response and the generation of memory
Source: PLoS Pathog. 2017 Aug 21;13(8):e1006544. doi: 10.1371/journal.ppat.1006544 (PMC5578684; doi:10.1371/journal.ppat.1006544)
Supplement: S6 Fig — (PDF) [file ppat.1006544.s006.pdf]

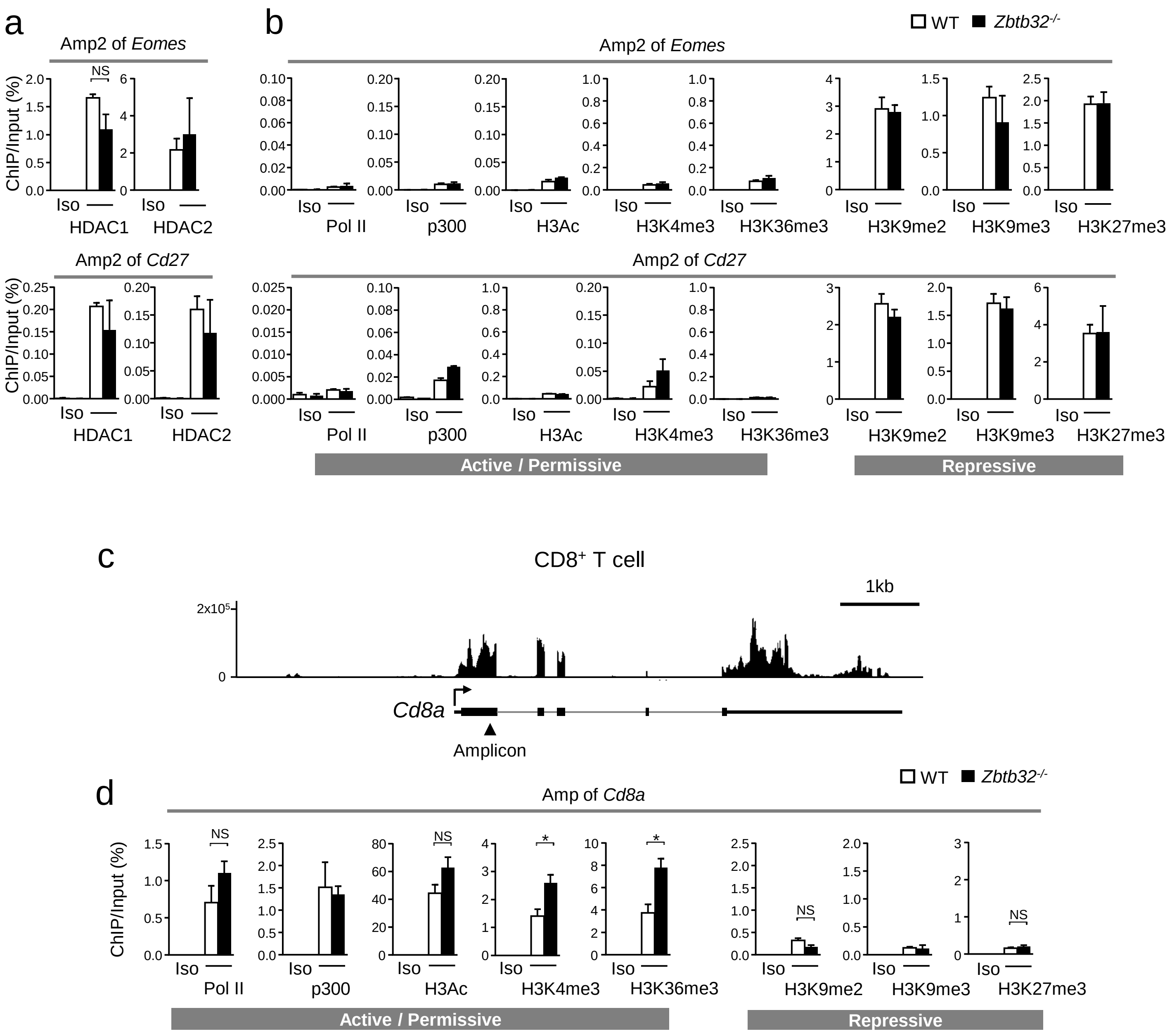

S6 Fig. Chromatin immunoprecipitation data from control regions of *Eomes*, *Cd27* and *CD8a* genes.

WT and *Zbtb32*$^{-/-}$ P14 splenocytes were transferred into recipients, which were then infected with LCMV-Armstrong. Chromatin was prepared at day 7 post-infection. ChIP assays were performed with the indicated antibodies and ChIP eluates were amplified by Q-PCR for target gene loci.

(a) The enrichment of HDAC1 and HDAC1 on negative control regions (Amp2) of *Eomes* (upper) and *Cd27* (lower) genes.

(b) ChIP for Pol II, p300 or modified histone H3 on negative control regions (Amp2) of *Eomes* (upper) and *Cd27* (lower) genes.

(c) RNA-Seq profile on *Cd8a* gene locus in naïve CD8$^+$ T cells. The original sequence data were obtained from the ImmGen consortium website (www.immgen.org) and visualized in the UCSC genome browser. The PCR amplicon (Amp) for the ChIP assays is indicated. (d) ChIP for Pol II, p300 or modified histone H3 on the *Cd8a* gene locus. Iso, isotype control antibody. Data are a compilation of three independent experiments; error bars represent the SEM.
