# Supplementary material for: Transient expression of ZBTB32 in anti-viral CD8+ T cells limits the magnitude of the effector response and the generation of memory
Source: PLoS Pathog. 2017 Aug 21;13(8):e1006544. doi: 10.1371/journal.ppat.1006544 (PMC5578684; doi:10.1371/journal.ppat.1006544)
Supplement: S7 Fig — (PDF) [file ppat.1006544.s007.pdf]

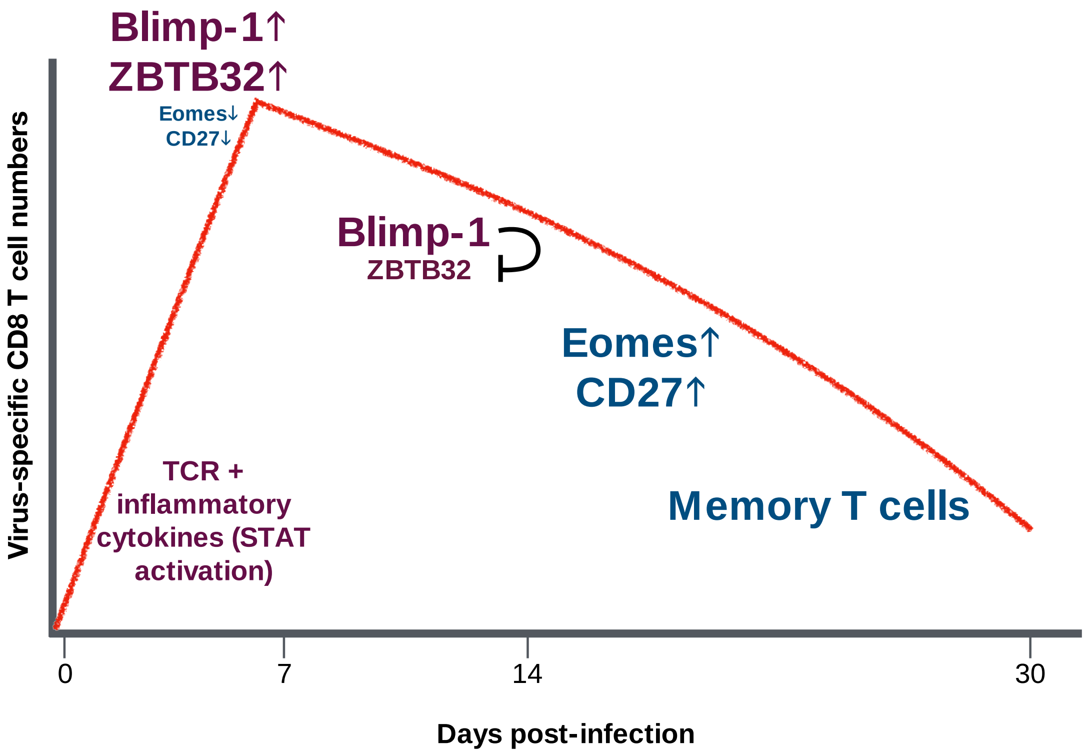

S7 Fig. Model for CD8+ effector versus memory T cell regulation by ZBTB32 and Blimp-1. Following acute virus infection, TCR stimulation plus signaling by inflammatory cytokine signals induce Blimp-1 and ZBTB32 expression in virus-specific CD8+ T cells. Together, these factors act to repress expression of memory T cell genes, such as *Eomes* and *Cd27*. As the virus is cleared, Blimp-1 represses expression of ZBTB32. In the absence of ZBTB32, *Eomes* and *Cd27* are no longer repressed. The upregulation of these proteins promotes long-lived memory cell generation.
