# Supplementary material for: Transient expression of ZBTB32 in anti-viral CD8+ T cells limits the magnitude of the effector response and the generation of memory
Source: PLoS Pathog. 2017 Aug 21;13(8):e1006544. doi: 10.1371/journal.ppat.1006544 (PMC5578684; doi:10.1371/journal.ppat.1006544)
Supplement: S8 Fig — (PDF) [file ppat.1006544.s008.pdf]

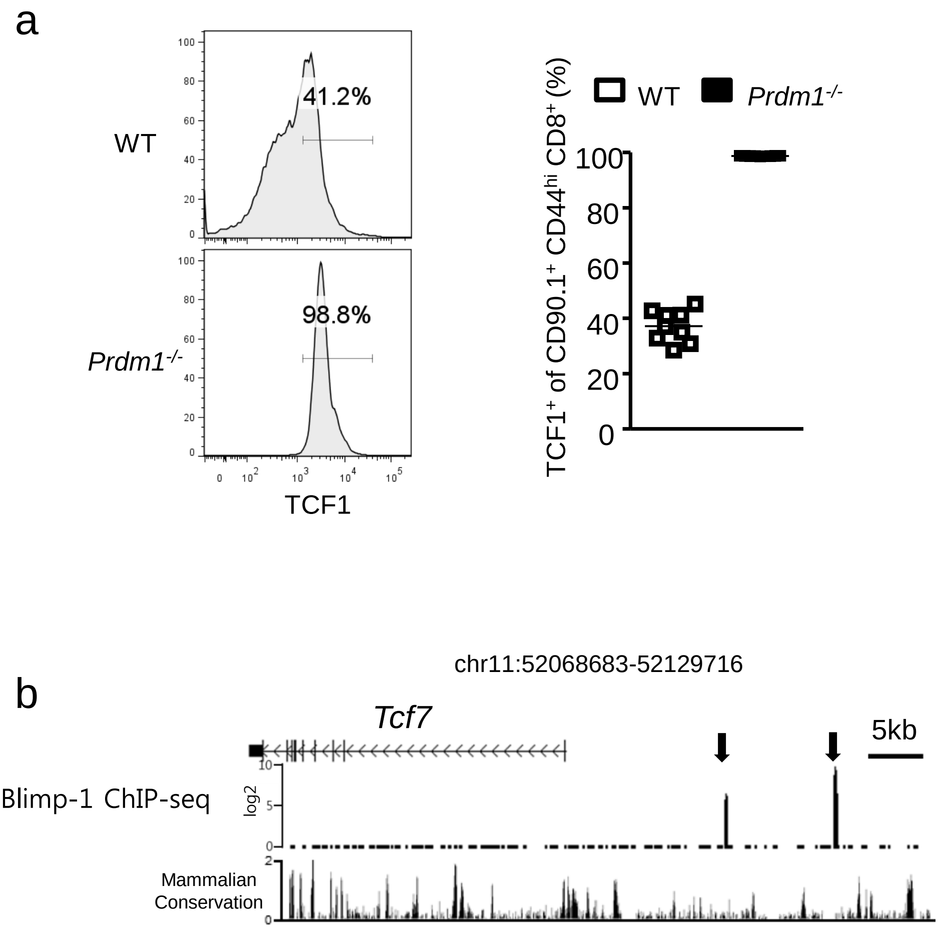

S8 Fig. Blimp-1 regulates TCF1 expression in CD8$^+$ T cells.

(a) WT or *Prdm1*$^{-/-}$ P14 splenocytes (5x10^4, CD90.1$^+$) were adoptively transferred into CD90.2$^+$ recipient mice followed by LCMV-Armstrong infection. At day 10 post-transfer and infection, P14 CD90.1$^+$ cells were analyzed for TCF1 expression by intracellular staining followed by flow cytometry, and a compilation of data is shown at right.
(b) Identification of Blimp-1 binding sites on the *Tcf7* gene. The scale bar indicates the relative kb scale and the numbers on the right display the magnitude of sequence enrichment on a log2 scale. Peaks were identified with Cisgenome 2.0, and all enrichment data on Blimp-1 binding sites are shown in the ChIP-seq tracks (15). Placental mammalian basewise conservation by PhyloP for the *Tcf7* gene locus is displayed below.
