# Supplementary material for: Transient expression of ZBTB32 in anti-viral CD8+ T cells limits the magnitude of the effector response and the generation of memory
Source: PLoS Pathog. 2017 Aug 21;13(8):e1006544. doi: 10.1371/journal.ppat.1006544 (PMC5578684; doi:10.1371/journal.ppat.1006544)
Supplement: S9 Fig — (PDF) [file ppat.1006544.s009.pdf]

Primers for Quantitative RT-PCR

Gene	Primer	Sequence	Gene	Primer	Sequence
Prdm1	Sense	5’-GAC GGG GGT ACT TCT GTT CA-3’	*Irf4*	Sense	5'-CAC AGC TCA TGT GGA ACC TCT-3'
	Anti-sense	5’-GGC ATT CTT GGG AAC TGT GT-3’		Anti-sense	5'-GCG GTG GTA ATC TGG AGT GG-3'
Eomes	Sense	5’-GGG ACA ACT ACG ATT CAT CCC A-3’	*Irf8*	Sense	5'-CGT GGA AGA CGA GGT TAC GCT G-3'
	Anti-sense	5’-GCA CGG TTC TCT CAC CGT TA-3’		Anti-sense	5'-GCT GAA TGG TGT GTG TCA TAG GC-3'
Zbtb32	Sense	5’-CCC ACT CCA GGA TCT TTT CCC-3’	*Bcl6*	Sense	5'-CTG CAG ATG GAG CAT GTT GT-3'
	Anti-sense	5’-TGA CTC ACA CAG GTT GCC AG-3’		Anti-sense	5'-CAC CCG GGA GTA TTT CTC AG-3'
Tbx21	Sense	5'-CAA CAA CCC CTT TGC CAA AG-3'	*Tcf7*	Sense	5'-CCC CGA TCT CTC TGG ATT TT-3'
	Anti-sense	5'-TCC CCC AAG CAG TTG ACA GT-3'		Anti-sense	5'-GGC AGG GAA GTG CTG TCT AT-3'
Id2	Sense	5'-ACC AGA GAC CTG GAC AGA AC-3'	*Lef1*	Sense	5'-CAG CCT GTT TAT CCC ATC AC-3'
	Anti-sense	5'-AAG CTC AGA AGG GAA TTC AG-3'		Anti-sense	5'-TCC TGT TTG ACC TGA GGT GT-3'
Id3	Sense	5'-GAC TCT GGG ACC CTC TCT C-3'	*Cd7*	Sense	5'-AGA TCA CCT GGA TTT GGG CG-3'
	Anti-sense	5'-ACC CAA GTT CAG TCC TTC TC-3'		Anti-sense	5'-CTG GTG TAC GTC TTG GGC AT-3'
Irf1	Sense	5'-TAA CTC CAG CAC TGT CAC CGT G-3'	*Bcl2*	Sense	5'-CTG GAA ACC CTC CTG ATT TT-3'
	Anti-sense	5'-TAT GCC TAT CCC AAT GTC CCC-3'		Anti-sense	5'-AAA TAT TTC AAA CGC GTC CA-3'

ChIP Amplicon Primers for Quantitative PCR

Gene	Amplicon	Primer	Sequence
Zbtb32	Amplicon A	Sense	5’-GAC CTT GAC CCC TTG AGC AG -3’
		Anti-sense	5’-GGA AGC CTG TGG TGG TCT TC -3’
	Amplicon B	Sense	5’-TCG CAG GCT ATC TCT CTC TGT-3’
		Anti-sense	5’-GCT ACT TCC TCT CAC AAG GAG G-3’
	Amplicon C (negative control)	Sense	5’-ACC TTG CTG TAG ATG GGT GC-3’
		Anti-sense	5’-GAC AAG GGG AAC CGA AGA GG -3’
Eomes	Amplicon 1	Sense	5'-CCT GGT GAG CTC GGT GAA C-3'
		Anti-sense	5’-GAG ACT GCC CGG AAA CTT CT-3’
	Amplicon 2 (negative control)	Sense	5’-AAC GCA ACA GGG AGA GGA AG-3’
		Anti-sense	5’-CCC ATG GGG TCT CTG AAC AC-3’
Cd27	Amplicon 1	Sense	5’-GAG CTG AGG TCA CAG GTT CC-3’
		Anti-sense	5’-AAA GCG GCA CAC ACC AAT TC-3’
	Amplicon 2 (negative control)	Sense	5’-GCA GGT ATG GAG TTT GGG GT-3’
		Anti-sense	5’-GAC ATG TGC CTG TCT GAG GT-3’
Il2ra	Amplicon 1	Sense	5’-TCG GAG AGG GAT TCG GTA GCT TGA-3’
		Anti-sense	5’-TGA TAG CCT GCT GCT CAG AAC TGG G-3’
	Amplicon 2 (negative control)	Sense	5’-TTA CAG CAG TGC CTC CCT TG-3’
		Anti-sense	5’-GGG AGT GAG TGG GGT TAG GA-3’
Cd8a	Amplicon	Sense	5’-CTG CCA TGA GGG ACA CGA AT-3’
		Anti-sense	5’-GAC TGG CAC GAC AGA ACT GA-3’

S9 Fig. Primers and ChIP Amplicon primers for PCR and quantitative PCR
