# Supplementary material for: Transient expression of ZBTB32 in anti-viral CD8+ T cells limits the magnitude of the effector response and the generation of memory
Source: PLoS Pathog. 2017 Aug 21;13(8):e1006544. doi: 10.1371/journal.ppat.1006544 (PMC5578684; doi:10.1371/journal.ppat.1006544)
Supplement: S10 Fig — (PDF) [file ppat.1006544.s010.pdf]

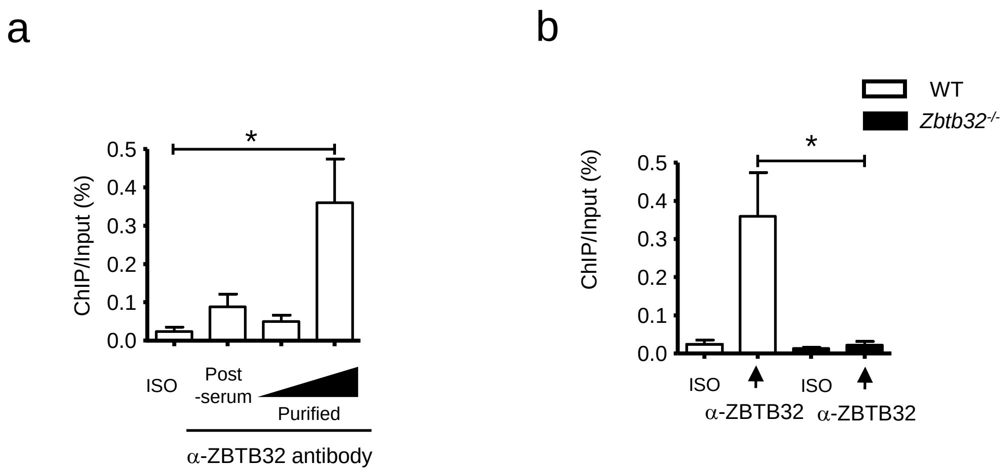


**S10 Fig. Titration of ZBTB32 antibody**

WT and *Zbtb32*$^{-/-}$ mice were infected with LCMV-Armstrong. CD44$^{hi}$ CD8$^{+}$ T cells were isolated at day 7 post-infection, and chromatin was prepared. Purified ZBTB32-specific antibodies as described in supplemental materials and methods were used for ChIP assays (a,b). ChIP eluates were amplified by Q-PCR for the *Eomes* gene locus. Iso, isotype control antibody.
